# Impact of public health and patient‐centered prevention strategies on periodontitis and caries as causes of tooth loss in high‐income countries

**DOI:** 10.1111/prd.12592

**Published:** 2024-09-25

**Authors:** Thomas Kocher, Peter Meisel, Sebastian Baumeister, Birte Holtfreter

**Affiliations:** ^1^ Department of Restorative Dentistry, Periodontology and Endodontology University Medicine Greifswald Greifswald Germany; ^2^ Institute of Health Services Research in Dentistry University of Münster Münster Germany

**Keywords:** caries, cohort, DMF‐T, edentulism, periodontitis, public health, tooth loss

## Abstract

In high‐income countries, the oral health of the population is influenced by public health interventions, widespread use of oral care products, dental practice measures, and the cost of dental treatment. We compiled information on changes of the prevalence of proximal and upstream determinants of periodontitis, caries, and tooth loss over the last three decades to outline their potential effects on changes of oral health during this period. Information was retrieved from repeated cross‐sectional studies and from published literature. While both the prevalence of edentulism and the number of missing teeth (from the DMF‐T index) decreased, the number of sound teeth as well as the total number of teeth increased. The prevalence of severe periodontitis was unchanged, whereas the prevalence of periodontal health and moderate periodontitis may have increased to a minor extent. Concerning oral health risk factors, the proportion of individuals with tertiary education increased, while smoking prevalence declined. More and more people used oral care products. Whether one reimbursement system worked better than another one in terms of tooth retention could not be elucidated. In tooth retention, population‐wide use of fluoridated toothpastes had the greatest impact. To some extent, the higher number of teeth present may be related to the more frequent use of interdental cleaning aids and powered toothbrushes. Since there was no decrease in severe periodontitis in most cohorts, periodontal interventions probably contributed little to improved tooth retention.

## 1 INTRODUCTION

In high‐income countries, the oral health of the population is influenced by public health interventions such as smoking bans in public places, by the widespread use of oral health products from consumer industry and by interventions in dental practices.^1^ In this review, we try to disentangle the influence of these different interventions and measures on oral health, based on a literature review and on the compilation of data from repeated population‐based cross‐sectional studies.

Public health is concerned with protecting the health of entire populations in contrast to clinical activities, which focus primarily on treating sick individuals. In his ground‐breaking book “Strategy of preventive medicine”, Rose showed that prevention strategies, that offer health benefits to the population, may have little benefit to the individual.^2^ The distribution of disease forms a continuum with healthy and severely ill individuals at the extreme ends of the distribution. Within a population, the majority has mild or moderate disease severity and, thus, contribute more cases to the burden of disease than the severely ill individuals, who present only a small number with extreme risks. Interventions targeting the risk determinants of the whole population can shift the risk curve to the left, thereby reducing the disease burden of the whole population. Such population‐wide, albeit public health interventions, are more effective for highly prevalent diseases than interventions targeting only the high‐risk groups, simply because patient‐centered medicine focuses on treating individuals with a high‐risk profile and severe disease, often neglecting individuals with mild or moderate disease.

Applying public health thinking to oral health means focusing on upstream determinants or interventions that target whole populations. In recent decades, there have been numerous calls for public health interventions to prevent periodontitis together with other noncommunicable diseases (cardiovascular disease, diabetes, chronic respiratory disease, and cancer) by focusing on common risk factors of noncommunicable diseases.^3^, ^4^, ^5^ An extensive literature review found many concepts, but only one program was implemented.^6^ In Japan, a population‐based public awareness campaign was implemented to retain at least 20 teeth in 80‐year‐olds. This campaign led to improved oral health.^7^ In contrast to this often demanded and requested public health approach, the current dental treatment approach in Western societies is heavily influenced by the biomedical etiology paradigm, which focuses on cavity filling and plaque control through chairside intervention and is concentrated on the individual. It is highly controversial, whether this traditional clinical approach has any impact on prevalences of caries and periodontitis at the population level.^5^ According to Sheiham “The isolated, compartmentalized and individualistically focused approach will never effectively promote oral health in all sections of the community”.^8^

As most health care systems around the world struggle with rising costs, it is important for stakeholders and health policy decision‐makers to use resources efficiently. Classical public health determinants, such as socio‐economic status (SES), smoking, diet, diabetes, and their impact on periodontal health will not be discussed here in detail, as comprehensive reviews have recently been published.^9^, ^10^, ^11^, ^12^, ^13^ Instead we attempt to rate the extent, to which changes in the proximal and upstream determinants may have altered the burden of oral disease within populations. This approach differs from many other reviews that have focused on the impact of specific determinants of periodontitis and have not considered that the prevalence of the determinants may also have changed over time. We consider periodontitis and tooth loss as outcomes. Since tooth loss is also the ultimate consequence of caries, we also briefly address its population burden.

In many high‐income countries, caries prevalence has decreased in children, the number of teeth in adults has increased and edentulism has decreased in older people. This raises the question of whether the higher number of teeth is only due to the decline in caries among adults and seniors,^14^ or whether a decline in periodontitis has also contributed to this trend.^15^ Although the recent Global Burden of Disease study reported a decrease of the severe periodontitis prevalence by 14.3% between 1990 and 2017,^16^ a recent review concluded, that “given the many methodological challenges, no firm conclusions with regard to a declining trend in periodontitis prevalence can be drawn at this time”.^17^ When available, we based our review on repeated cross‐sectional studies as the primary source of information, because they have identical sampling procedures, recording protocols, etc.^15^ and we restricted our review to high‐income countries, for which most of the literature is available.^5^, ^6^, ^18^ Considering preventive habits or interventions, our conclusions will concern effectiveness (effectivity), which denotes outcomes achieved under everyday conditions in the population and not efficiency (efficacy under ideal conditions) with individual patients in academic or specialist settings.

The focus of this review is on the impact of periodontitis as a cause of tooth loss. However, tooth loss data cannot be understood without considering caries. In the relevant paragraphs, we refer to specific reviews.

## 2 CAUSAL NETWORK FOR TOOTH LOSS

**FIGURE 1:**
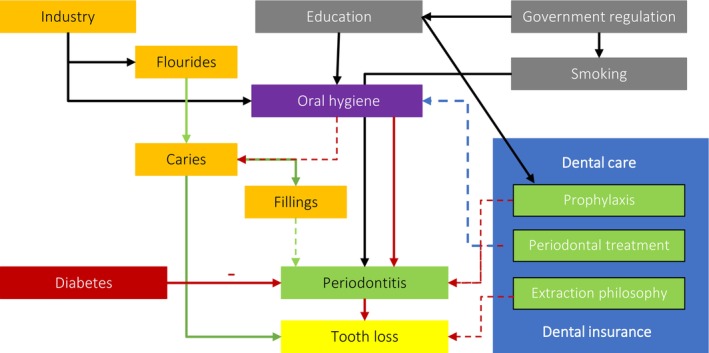
Causal network of tooth loss with three major determinants as drivers: government regulation, industry, dental care, dental insurance and diabetes.

We have created a very simple causal network of tooth loss to show how different determinants might interact and affect periodontitis and tooth loss with 5 major determinants as drivers: government, industry, dental care, dental insurance, and diabetes (Figure 1). We briefly outline and justify this model with examples:

- In high‐income countries, the economy demands a more educated workforce. Education and, subsequently, income are the most important SES factors. Both are associated with better financial potential, better access to care, better self‐perception and improved oral hygiene (lead: government regulation).
- Smoking cessation is mainly driven by legislation and indirectly affects the prevalence of periodontitis (lead: government regulation).
- Access to the dentist and reimbursement of treatment depends on the remuneration system and reimbursement of treatment cost. In the dental office, the dentist and its team address oral hygiene and perform prophylaxis sessions, which in turn improves the periodontal status. Successful periodontal treatment results in reduced tooth loss. Over the last few decades, the philosophy of periodontal treatment has changed significantly with regard to the extent to which periodontally compromised teeth can be preserved (lead: dental care, remuneration system (under government regulation)).
- The nearly 100 percent use of fluoridated toothpastes in the general population has led to a decline in caries and caries‐related tooth loss. One consequence of the decrease in caries is the reduction of fillings, which may result in less iatrogenic damage to the periodontium (lead: industry).
- Promotion of oral care products by the industry and dental team can improve consumer awareness of periodontal disease and lead to better oral hygiene (lead: industry, dental care).
- However, an unhealthy diet (highly processed foods, high consumption of animal products, refined grains, sugars, saturated fats, and trans‐fatty acids) affects oral health directly and indirectly through diabetes (lead: industry, dental team).

Based on this simplified causal network of tooth loss we have attempted to compile information on changes of the prevalence in the proximal and upstream determinants of periodontitis, and tooth loss over the last three decades and to outline their potential effects on caries, periodontitis, and tooth loss. We have not conducted a formal analysis. Therefore, our conclusions on trends and shifts are only tentative.

## 3 EVIDENCE DERIVED FROM RANDOMIZED CONTROLLED TRIALS, QUASI‐EXPERIMENTAL AND OBSERVATIONAL STUDIES

The gold standard for assessing policies, treatment effects, informing treatment decisions, and revealing negative side effects of treatments is the randomized controlled trial (RCT). The key advantage of a randomized trial is that treated and untreated groups are expected to be exchangeable and that any differences in outcomes can be attributed to treatment and not to prognostic differences between groups. Another reason why trials support causal inference is that the start of follow‐up is clearly defined at time of randomization. When trials involve sustained treatment strategies, they often show lack of adherence and dropout, which makes them subject to post‐randomization confounding and selection biases. Hence, there is a growing interest in using observational data to evaluate the effectiveness and safety of preventive and clinical interventions and policies. Many policy evaluations and efficacy and safety studies therefore rely on population‐based cohorts, electronic medical records, and administrative claims. Efficacy and safety inferences from observational data are, however, challenging, because observational studies are prone to confounding due to lack of randomization. Additionally, incorrect study design can induce self‐inflicted bias. For example, by including prevalent users of a treatment, observational studies are prone to selection bias. This occurs because the treatment group includes individuals who used the treatment before start to follow‐up. A problem with all observational studies is residual confounding, which refers to the presence of confounding factors that are not adequately accounted for in the analysis of a study. For example, in studies investigating the effect of powered toothbrush use on periodontal health, the socio‐economic status (SES) must be adjusted for in the analysis, not because the relationship between SES and periodontal health is being investigated (which is known), but because powered toothbrush use may differ across different levels of SES. Participants with higher SES may have higher health literacy or more money to buy a powered toothbrush. This means that if the SES is not included in the analysis, there may be an artefactual relationship between powered toothbrush use and periodontal health due to residual confounding by SES.

The limitations of observational data can, in part, be overcome by designing the study as a quasi‐experiment or natural experiment.^19^, ^20^, ^21^, ^22^ Quasi‐experiment or natural experiment studies are at the intersection between randomized trials and observational studies, and if properly conducted, provide the best alternative to trial evidence. Quasi‐experimental designs include regression discontinuity, interrupted time series, and difference‐in‐difference methods. Regression discontinuity and interrupted time series methods are applicable when there is an arbitrary discontinuity in the probability of being treated depending on the value of a third “forcing” variable. Examples of “forcing” variables include age, income, or class size. Individuals immediately above or below that cut point are expected to have equivalent potential outcomes, except for the effects of the sharp differences in treatment policies. Difference‐in‐difference methods combine regression discontinuity with one or more external comparison groups, which can account for other sources of variation at the discontinuity. Difference‐in‐difference is valuable when the date of change in a policy affecting treatment (e.g. mandatory school law changes) is used as a discontinuity. Designs referred to as regression discontinuity and difference‐in‐difference can be conceptualized and analyzed as instrumental variables.^22^ The instrumental variable approach exploits an exogenous factor – some program, policy, or arbitrary variation – that creates differences in the chance of receiving treatment between groups of individuals with otherwise similar potential outcomes and the exogenous factors must have no other mechanism to influence the outcome except via the treatment under consideration. Sources of instruments include changes in policies at unprecedented times, months or quarter of birth, or biological chance, such as sex of a child or random allocation of genetic variants at conception. When there are no quasi‐experimental devices available, evidence must be derived from, and clinical and policy decisions must be based on rigorously designs and analyzed confounder‐controlled prospective observational studies. In such studies effects are estimated by comparing outcomes of exposed/treated to unexposed/untreated individuals and potential imbalances in confounding variables are addressed by regression‐adjustment, stratification, weighting, or equivalent methods.

Repeated cross‐sectional studies provide further insight into trends in oral conditions and related change in exposures and treatments. However, they cannot distinguish between age, cohort, and period effects. The compilation of trend data is based on representative national or regional surveys in high‐income countries that have conducted repeated surveys to track changes in the same determinants and outcomes over time (Table 1).

**TABLE 1:** Overview of included studies.

Study/Country/Region/Abbreviation/ Reference	Study period	Number of participants (response)/Age range in years	Recording of the variables used/Protocol and probe	Outcomes used in this review
Adult Oral Health Australia National study Abbreviated: Australia 23, 24, 25	1987–1988 2004–2006 2017–2018 1987–1988 2004–2006 2017–2018	Examined 14 430 55 005 (43.7%)^a^ 5022 (33.6%)^a^ Interviewed 16 897 28 812 (58%)^b^ 15 731 (40%)^b^ Age range 15+	Edentates (interview) Tooth count (including 3^rd^ molars, excluding edentates) DMFT: Coronal caries (excluding 3^rd^ molars): decayed (visual manifestation cavitation, periodontal probe detection of hardness), filled, sound, missing Recording protocol periodontitis: 2004–2006 and 2017–2018: all teeth present, three sites (mesio‐, mid‐ and distobuccal), excluding 3^rd^ molars Probe with 2 mm markings CDC/AAP case definition	% Edentates (interview) Number of teeth, DMFT (including 3^rd^ molars, excluding edentates) Decayed teeth Filled teeth Sound teeth (calculated) CDC/AAP case definition (moderate and severe reported together) No/mild cases All variables were recalculated with edentates as own category.
New Zealand Oral Health Survey (NZOHS) National study Abbreviated: NZ 23, 27	1988 2009 1988 2009	Examined 1485 (51%–61%)^c^ 2209 (55%)^d^ Interviewed 1777 (71%–80%) 3475 (70%) Age range 20–24/35–44/65–74	Edentate (interview) Oral examination in dentates only. Tooth count (including 3^rd^ molars, excluding edentates) DMFT: Coronal and root caries (including 3^rd^ molars), decayed (cavitation), filled, missing Recording protocol periodontitis: 1988: CPITN, six sites at 10 index teeth WHO probe (markings 3.5, 5.5, 8.5, 11.5 mm) 2009: PPD, CAL, BOP at three sites (mesio‐ and midbuccal, distolingual) of all teeth excluding 3^rd^ molars; converted to CPI NHANES probe (2 mm markings) Recording protocol harmonized to the one used in 1998.	% Edentate (interview) Number of teeth, DMFT (including 3^rd^ molars, excluding edentates) DMFT Decayed teeth Filled teeth Sound teeth (calculated) CPI 0, 1, 2 CPI 3 CPI 4 All variables were recalculated with edentates as own category. Cohorts (20–24,35–44, 65–74) were merged with weights according to number of examined persons.
National Survey of Dental Disease, Japan National study Abbreviated: Japan ^28^	1993 1999 2005 2011 2016	Examined 7246 (58%) 5528 (46%) 3867 (31%) 3605 (n.a.) 3329 (n.a.) Age range 20–80+	Edentate (examination) Tooth count (including 3^rd^ molars, including edentates DMFT: (including 3^rd^ molars, including edentates), decayed, filled, sound, missing Protocol not reported Recording protocol periodontitis: PPD, BOP, calculus Six sites (mesio‐, mid‐, distobuccal, mesio‐, mid‐, distolingual) on index teeth (16, 17, 11, 26, 27, 36, 37, 31, 46, 47) WHO probe	% Edentate (examination) Number of teeth, DMFT (including 3^rd^ molars, excluding edentates) Decayed teeth Filled teeth Sound teeth CPI 0, 1, 2, 3,4 All variables were recalculated with edentates as own category.
Korea National Health and Nutrition Examination Survey (KNHANES) National study Abbreviated: South Korea 29, 30, 31, 32	1st (1998) 2nd (2001) 3rd (2005) 4th (2007 2009) 5th (2010–2012) 6th (2013–2015) 7th (2016–2018)	Examined 27 745 (n.a.) 27 400 (n.a.) 7597 (70.2%) 23 632 (74.5%) 24 173 (76.5%) 21 724 (74.1%) 23 162 (73.1%) Age range 20+	Edentates (examination; percentage from graph) DMFT: not reported^g^ Recording protocol periodontitis: PPD Six sites (mesio‐, mid‐, distobuccal, mesio‐, mid‐, distolingual) on index teeth (16,17, 11, 26, 27, 36, 37, 31, 46, 47) WHO probe	% Edentates (examination) Number of teeth (excluding 3^rd^ molars)^33^ CPI 3 and 4 reported together CPI 0, 1, 2 calculated All variables were recalculated with edentates as a separate category.
Adult Dental Health Survey (ADHS) England, Wales and Northern Ireland Restricted to England, because not all data were available across complete cohort Abbreviated: England 34, 35	1988 1998 2009 1998 1998 2009	Examined 2500 (n.a.) 2186 (72%)^a^ 6470 (61%)^a^ Interviewed 3751 (n.a.) 3436 (92%)^b^ 9660 (92%)^b^ Age range 1988, 1998: 16–75+ 2009: 6–85+	Edentates (interview) Oral examination in dentates only. Tooth count (including 3^rd^ molars) DMFT: Coronal and root caries (including 3^rd^ molars): decayed (visual manifestation and cavitation into dentine), filled, sound, missing Recording protocol periodontitis: 1998, 2009: BOP, PPD, CAL Two sites (mesial and distal; buccally upper teeth, lingually lower teeth); all teeth; worst score in each sextant recorded CAL recorded only in subjects ≥55 years old. WHO probe (markings 3.5, 5.5, 8.5, 11.5 mm)	% Edentates (interview) Number teeth, DMFT (including 3^rd^ molars, excluding edentates) Decayed teeth Sound teeth Filled teeth Sound teeth PPD ≤3 mm PPD ≥4 mm PPD ≥6 mm All variables were recalculated with edentates as a separate category.
Encuesta de Salud Oral en España National study Abbreviated: Spain 36, 37, 38, 39	1993 2000 2005 2010 2015 2020	Examined 1013 (n.a.) 1073 (n.a.) 1080 (n.a.) 998 (n.a.) 1165 (93.1%) 1106 (75.6%) Age range 35–44/65–74	Edentate (examination) Tooth count (excluding 3^rd^ molars, including edentates) DMFT: Coronal caries (excluding 3^rd^ molars, cavitation, WHO criteria ^40^), sound, filled, missing Recording protocol periodontitis: PPD, BOP, calculus Six sites (mesio‐, mid‐, distobuccal, mesio‐, mid, distolingual) on index teeth (16, 17, 11, 26, 27, 36, 37, 31, 46, 47) WHO probe	% Edentate (examination) Number of teeth (excluding 3^rd^ molars, including edentate) DMFT (excluding edentates) Decayed teeth Filled teeth Sound teeth (calculated) CPI 0, 1, 2 CPI 3 CPI 4 All variables were recalculated with edentates as a separate category. Data of age cohorts (35–44 and 65–74) were merged and weights were applied.
Deutsche Mundgesundheitsstudie (DMS) (German Oral Health study) Germany National study Abbreviated: Germany ^41^	1997 (DMS III) 2005 (DMS IV) 2014 (DMS V)	Examined 2022 (56.1%) 3642 (54.0%) 2108 (49.0%) Age range 35–44/65–74	Edentates (oral examination, including 3^rd^ molars) Tooth count (excluding 3^rd^ molars, including/excluding edentate) DMFT: Coronal caries (excluding 3^rd^ molars), decayed (visual manifestation and cavitation), filled, missing teeth (including edentatesHArmnized) Recording protocol periodontitis: DMS III: BOP, CAL, PPD 2 sites (mid‐ and mesiobuccal) on teeth of 1^st^ and 4^th^ quadrant, excluding 3^rd^ molars DMS IV: BOP, CAL, PPD 3 sites (midbuccal, mesiobuccal and distolingual) on twelve index teeth (17, 16, 11, 24, 26, 27, 47, 46, 44, 31, 36, 37) DMS V: BOP, CAL, PPD 3 sites (midbuccal, mesiobuccal and distolingual) on twelve index teeth (17, 16, 11, 24, 26, 27, 47, 46, 44, 31, 36, 37), WHO probe Harmonized periodontal recording protocol: 2 sites at teeth 17, 16, 11, 47, 46, 44	% Edentates (examination) Number of teeth, DMFT (excluding 3^rd^ molars, excluding edentates) Decayed teeth Filled teeth Sound teeth (calculated) CPI 0, 1, 2 CPI 3 CPI 4 All variables were recalculated with edentates as a separate category. Cohorts (35–44 and 65–74 years) were merged and weights were applied.
Study of Health in Pomerania West Pomerania, Germany Regional study Abbreviated: Germany SHIP 42, 43	1997–2001 (SHIP‐START‐0) 2008–2012 (SHIP‐TREND‐0)	Examined 4308 (68.8%) 4420 (50.1%) Age range 1997–2001: 20–81 2008–2012: 20–83	Edentates (Examination) Tooth count (excluding 3^rd^ molars, excluding edentates) DMFT: Coronal caries (excluding 3^rd^ molars, excluding edentates), decayed (cavitation), filled, missing, sound Recording protocol periodontitis: PPD, CAL, BOP PPD on 4 sites (mesio‐, mid‐ and distobuccal, midlingual); half‐mouth, all teeth excluding 3^rd^ molars 1997–2001: PCP11 probe 2008–2012: PCP15 probe	% Edentates (examination) Number of teeth, DMFT (excluding 3^rd^ molars, excluding edentates) Decayed teeth Filled teeth Sound teeth PPD ≤3 mm PPD ≥6 mm As the DMFT was recorded half‐mouth, scores were doubled. All variables were recalculated with edentates as a separate category.
Jönköping Study Jönköping, Sweden Regional study Abbreviated: Sweden Jönköping 44, 45	1993 2003 2013	Examined 655 (72.0%)^h^ 589 (64.7%)^h^ 621 (51.7%)^i^ Age range 20/30/40/50/60/70/80	Edentate (Examination) Tooth count (excluding 3^rd^ molars) DMFT: Coronal caries (cavitation and x‐ray), (reported DF‐T and D‐S) filled (reported F‐S), clinical and radiographic examination for approximal lesions coronal Recording protocol periodontitis: PPD four sites; full‐mouth Hilming probe Bone loss on x‐ray: mesial and distal for each pre−/molar 20–50‐year‐olds: orthopantomogram and 6 bite wings; 60–80‐year‐olds: orthopantomogram and a full‐mouth X‐ray status. Bone loss: Moderate: bone loss around most teeth <1/3 root length, Severe: bone loss around most teeth >1/3 or angular bony or furcation defects.	% Edentate (examination) Number of teeth, DFMT (excluding 3^rd^ molars, excluding edentates, DMFT recalculated from DMFS) Decayed teeth Filled teeth Sound teeth (calculated) No bone loss Moderate bone loss Severe bone loss All variables were recalculated with edentates as a separate category
Dalarna, Sweden Regional study Abbreviated: Sweden Dalarna 46, 47	2003 2008 2013 2003 2008 2013	Examined 1107 (72%)^a^ 1105 (61%)^a^ 1115 (50%)^a^ Interviewed n.a. 1130 (78%)^b^ n.a. Age range 35–85	Edentates (interview) Tooth count (including 3^rd^ molars) DMFT: Coronal caries (including 3^rd^ molars, excluding edentates), clinical and radiographic examination, caries (extending into dentine), filled, missing Recording protocol periodontitis: PPD: 2 sites (mesio‐ and distobuccal) including 3^rd^ molars Bone loss on x‐ray: premolar and molar on bite wings Moderate bone loss: distance CEJ to alveolar crest >2 mm, not exceeding one‐third of the length of the roots Severe bone loss: exceeding one sthird of the length of the roots	% Edentates (interview) Number of teeth, DMFT (including 3^rd^ molars, excluding edentates) Decayed teeth Filled teeth Sound teeth (calculated) No bone loss (calculated) Moderate bone loss Severe bone loss All variables were recalculated with edentates as a separate category
The National Health and Nutrition Examination Survey (NHANES) United States National study Abbreviated: USA 48, 49, 50	1988–1994 (NHANES III) 1999–2004 (NHANES 1999–2004) 2011–2016 (NHANES 2011–16) 1988–1994 1999–2004 2011–2016	Examined 10 992 (85.7%)^e^ 5794 (85.8%)^e^ 25 566 (64.9%)^e^ Interviewed 16 332 (80%)^f^ 15 332 (75%)^f^ 27 925 (68%)^f^ Age range 20–64	Edentate (examination) Tooth count (including 3^rd^ molars, excluding edentate) DMFT: Coronal caries (excluding 3^rd^ molars): decayed (cavitation, explorer detection of hardness), filled, sound, missing Recording protocol periodontitis: PPD, CAL, BOP NHANES III: 2 sites (mesio‐ and midfacial) in two randomly selected quadrants (one maxillary and one mandibular), excluding 3^rd^ molars; probe with 2 mm markings NHANES 1999–04: (mesio‐, mid‐ and distofacial); probe with 2 mm markings NHANES: 6 sites per tooth (mesio‐, mid‐, and distobuccal; mesio‐, mid‐, and distolingual) for all teeth, excluding 3^rd^ molars; probe with 2 mm markings Recording protocol harmonized to the one used in 1998.	% Edentate (examination) Number of teeth, DMFT (excluding 3^rd^ molars, excluding edentates) Decayed teeth Filled teeth Sound teeth (calculated) %PPD ≤3 mm %PPD 4 mm %PPD 5 mm %PPD ≥6 mm All variables were recalculated with edentates as a separate category.

^a^ Percentage of subjects with dental examination based on the number of dentate adults eligible for examination.

^b^ Percentage of subjects with dental interview based on the number of edentate and dentate adults eligible for interview.

^c^ For three age groups.^26^

^d^ Response rate for subjects with dental examination aged 18–75+ years, considering that the 2009 NZOHS was a follow‐up survey to the 2006/07 NZHS (Methodology Report for the 2009 New Zealand Oral Health Survey).^27^

^e^ Number of subjects with oral health examination relative to the number of sampled persons completing a Home Interview Questionnaire.^49^

^f^ https://wwwn.cdc.gov/nchs/nhanes/ResponseRates.aspx.^51^

^g^ Retrieved and recalculated from Figure 1 for each survey year.^52^

^h^ Calculated based on Hugoson et al.^53^

^i^ Calculated based on Norderyd et al.^44^

## 4 EFFECTIVENESS OF PUBLIC HEALTH MEASURES

Because the prevalence of upstream health determinants has changed substantially over the past three decades, we present their trend data from 1988 onward. In addition, the first waves of all repeated cross‐sectional studies cited in this review were conducted from 1988 onwards. In addition to education as a proxy for SES, we discuss smoking and diabetes as upstream determinants. Changes in upstream determinants take time to impact dental outcomes, e.g. the risk of periodontitis decreased linearly with time since smoking cessation. It takes about 11 years of smoking cessation for an ex‐smoker to no longer have a higher risk of periodontitis than a non‐smoker.^54^

### 4.1 Social inequality

SES is reflected by education, occupation, income, poverty, housing, social network, wealth and property, among other things, and shows a pronounced gradient in all industrialized societies. The higher the SES, the better the general health, the lower the burden of chronic diseases, and the higher the life expectancy.^55^ SES acts as an upstream determinant of general and oral health and is mediated through many proximal behavioral and biological pathways (smoking, diet, physical activity, oral hygiene habits, access and use of medical services, chronic inflammation). A landmark RCT clearly demonstrated the impact of preschool and school support on educational attainment and on health.^56^, ^57^, ^58^ Children from low‐income families were randomized to either a test group, which received cognitive and social support in preschool and school, or to a community control group. At the age of 21, the treatment group had higher academic scores and, interestingly, the preschool program had more impact than the school support. In their mid‐30s, the test group had a more favorable cardiovascular risk profile than the control group. This RCT impressively underlines the impact of education on general health and shows that benefits during childhood persist into adulthood.^56^, ^57^, ^58^ The authors concluded that “Early childhood interventions are an unexplored and promising new avenue of health policy”. The causal effect of education on health had also been elegantly demonstrated using US census data to estimate the impact of changes in compulsory schooling on mortality^59^ Birth cohorts before and after the change in school years were compared with life expectancy data. Each additional year of schooling led to a gain of 1.7 years in life expectancy at the age of 35.^60^ The results showed convincingly that investment in education leads to better health in later life.^61^

By and large, vulnerability and susceptibility to disease are concentrated at the lower end of the socio‐economic spectrum.^55^ As with many other chronic non‐communicable diseases, the social gradient is also evident in the prevalence and incidence of periodontitis and tooth loss.^11^, ^12^, ^62^ Inequalities in periodontitis and tooth loss vary widely between and within countries.^9^, ^63^ In all studies, all aspects of oral disease were more prevalent and more severe in economically disadvantaged persons.^11^, ^25^, ^35^, ^37^, ^41^, ^64^, ^65^, ^66^ While over the past decades, rates of advanced periodontitis have declined across all SES strata, changes were significantly more pronounced in college‐educated people than in people with no high school education (Figure 4).

**FIGURE 2:**
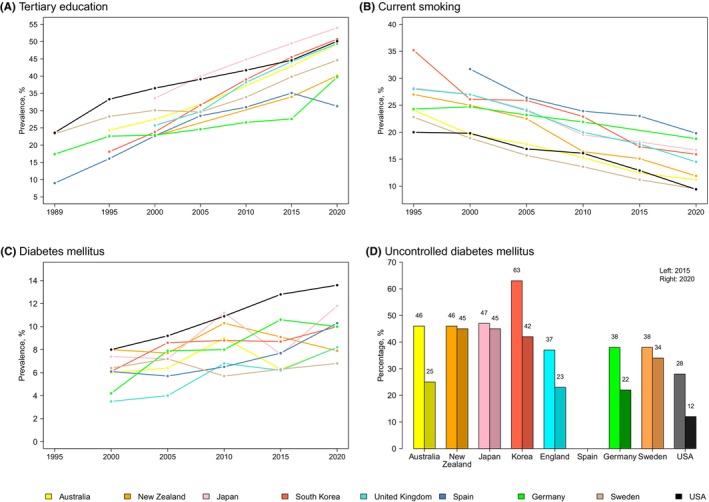
The most important determinants of periodontitis are socioeconomic status, smoking, and diabetes. Since data on these three determinants were not available in all selected cohorts, we accessed external registries (OECD and International Diabetes Federation). (A) The proportion of 25‐ to 55‐year‐olds in a population with a tertiary education (defined as those having completed the highest level of education, by age group. This includes both theoretical programs leading to advanced research or high skill professions such as medicine) increased from 10%–25% in 1989 and to 30%–55% in 2020.^69^ (B) During the same period, the prevalence of current smoking fell from 20%–35% to 14%–20%.^70^ (C) The diabetes prevalence increased from 4%–8% to 6%–14%.^71^, ^72^, ^73^, ^74^, ^75^ (D) Among diabetics, the proportion of well‐controlled diabetics increased in parallel.

**FIGURE 3:**
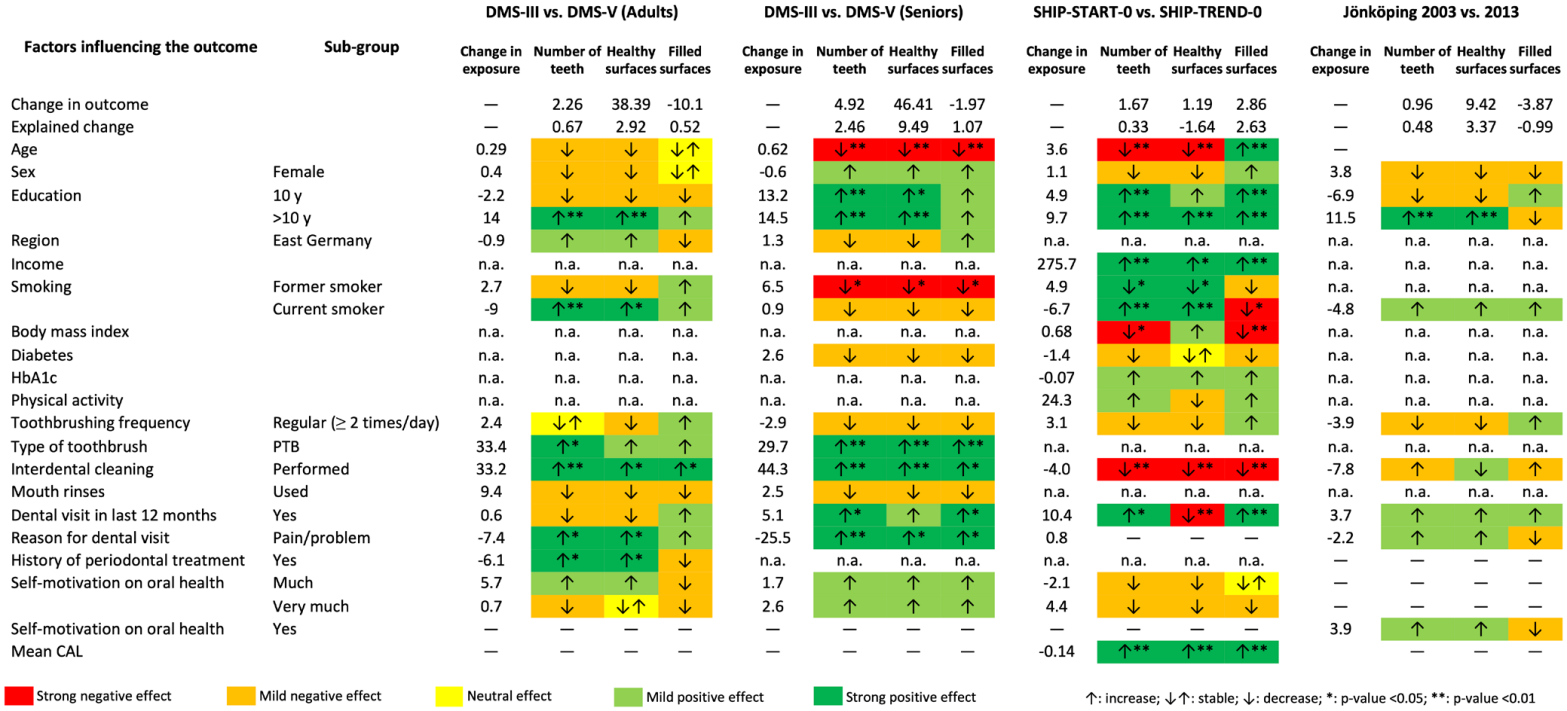
Overall trends of various determinants as well as their influence on the change of number of teeth present and the number of healthy or filled surfaces between two examinations were analyzed based on repeated population‐based cross‐sectional data (Germany DMS III‐DMS V; Germany SHIP‐START‐0–SHIP‐TREND‐0; Sweden Jönköping 2003–2013). Improvements in education and dental awareness brought a positive change in all outcomes. Education was the most important factor having both direct and indirect effects on the outcomes; powered toothbrushing and interdental cleaning were proximal determinants.^67^

Trends in globalization and the development of technology have changed the labor market's needs in high‐income societies, demanding more people with specialized skills. As a result, all affluent societies are experiencing a shift towards more people with higher, tertiary education. Over the last 25 years, the proportion of adults (25–55 years) with tertiary education has increased from around 10%–25% in 1995 to 25%–55% (Figure 2A). The shift towards higher education has also affected oral disease prevalences. Multivariable analysis of repeated cross‐sectional data (see Figure 3) from three population‐based studies (two German studies: Deutsche Mundgesundheitsstudien (DMS) [DMS III vs. DMS V], Studies of Health in Pomerania (SHIP) [SHIP‐START vs. SHIP‐TREND]; one regional Swedish study [Jönköping 2003 vs. 2013]; more information about the studies is provided in Table 1) showed that the shift towards a higher proportion of subjects with >10 years schooling was consistently associated with more teeth.^67^ SHIP‐TREND participants had 1.67 more teeth than their SHIP‐START‐0 counterparts examined 10 years earlier (Figure 3). In SHIP‐TREND, the proportion of subjects with >10 years of schooling was 9.7% higher than in SHIP‐START; their mean income had increased from 1233€ (standard deviation (SD) 642) to 1509€ (SD 765).^67^ The Oaxaca decomposition revealed that improvements in schooling and income explained 0.30 of the 1.67 more teeth in SHIP‐TREND‐0 than in SHIP‐START‐0. Between DMS III (1997) and DMS V (2014), the mean number of teeth in adults (seniors) had increased from 24.0 to 26.2 (14.5 to 19.4) and education >10 years had increased by 14% (14.5%). This increase explained 0.23 (0.38) of the 2.26 (4.92) more teeth in DMS V adults (seniors). Among individuals in the Jönköping‐2003 and the Jönköping‐2013 cohorts, the average number of teeth changed from 24.9 to 25.8, with the increased proportion of higher education (11.5%) explaining an additional 0.15 teeth. The authors concluded that education was the most important factor with direct and indirect effects on an increased number of teeth.^67^ In the United Kingdom, the duration of compulsory schooling increased by 0.62 years (95% CI: 0.41–0.84) between 1947 and 1972.^68^ Using reform in mandatory schooling as an instrumental variable, Matsuyama et al. showed that an additional year of education reduced the probability of edentulism by 9.1% (95% CI: 1.5–16.8) using three cross‐sectional datasets (2006–2007, 2010–2011, 2014–2015) of the English Longitudinal Study of Aging.^68^

**FIGURE 4:**
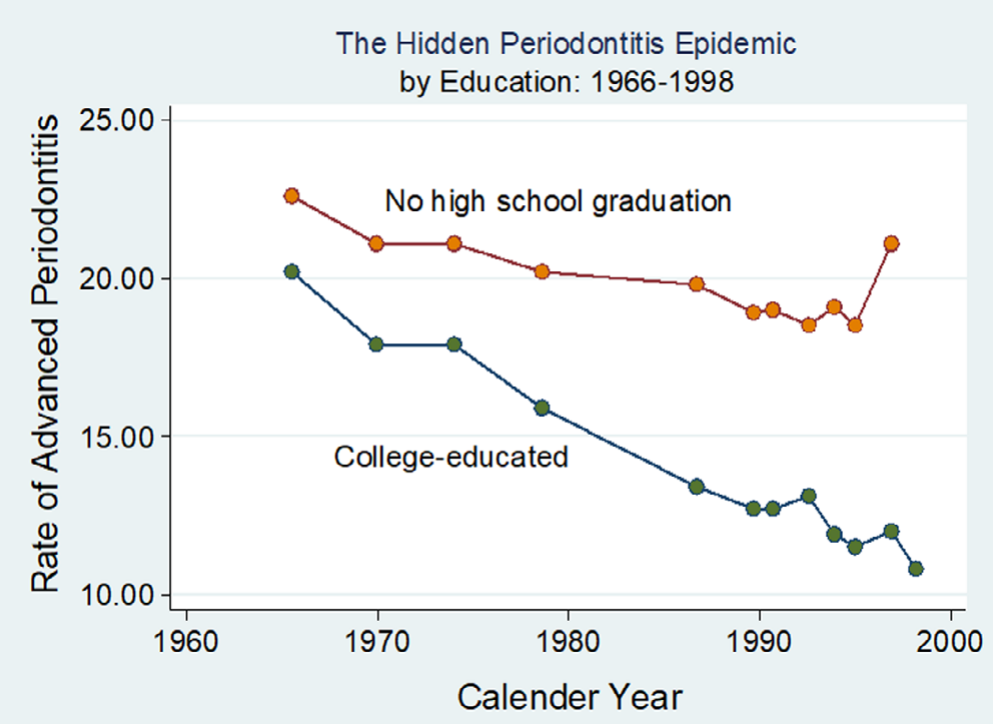
The impact of education on the incidence of periodontitis in the US (cases per 10 000 persons per year). The magnitude of the decreased periodontitis incidence depended on socio‐economic status. Between 1966 and 1998, in subjects with no high school education, the incidence decreased by only 8% in contrast to subjects with a higher education, whose periodontitis incidence decreased by 43% with an assumed relative risk of 6. Prevalence estimates of periodontitis was based on NHANES III and of smoking on the National Health Interview Survey data.^10^

**FIGURE 5:**
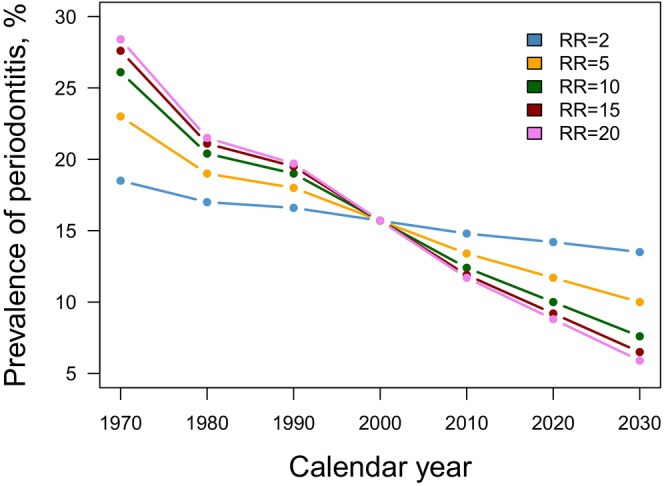
To illustrate the concept of population‐attributable fraction Bergström calculated the estimated prevalence of periodontitis in Sweden for the age bracket 40–70 years with smoking‐associated relative risks (RR) between 2 and 20. Prevalence rates were forecasted for 2010 to 2030. The smoking rate in Sweden declined from 44% in 1970 to 15% in 2010 and for an assumed 10‐fold smoking‐associated relative risk of periodontitis prevalence dropped from 26% to 12%. The population‐attributable fraction, estimating the portion of the disease that would have been avoided in the absence of smoking, was 80% in 1970 and 58% in 2010 assuming a 10‐fold relative risk. Smoking rates were taken from Swedish National Statistics on smoking habits and periodontal prevalence rates from the Jönköping studies.^76^

**FIGURE 6:**
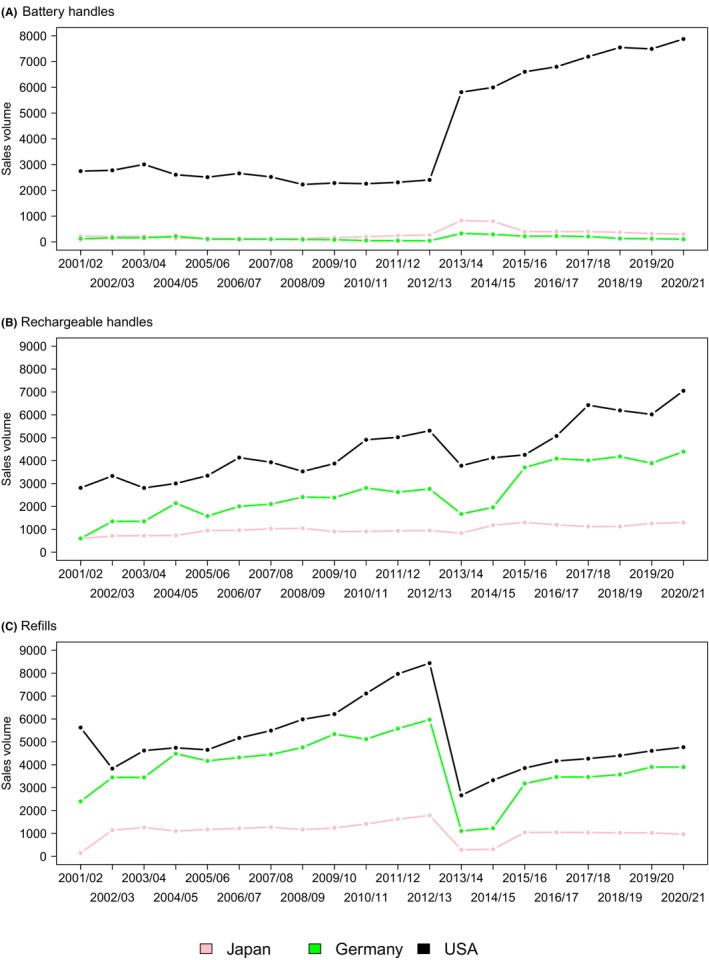
The graph shows the increase of electric toothbrushes over a period of 20 years in a company‐internal metric (sales volume, equalized volume unit, data provided by Procter & Gamble). In the USA, Japan and Germany, sales volumes for both battery (A) and rechargeable (B) toothbrushes increased markedly between 2001 and 2021, and in line the corresponding toothbrush attachments (refills, C).

**FIGURE 7:**
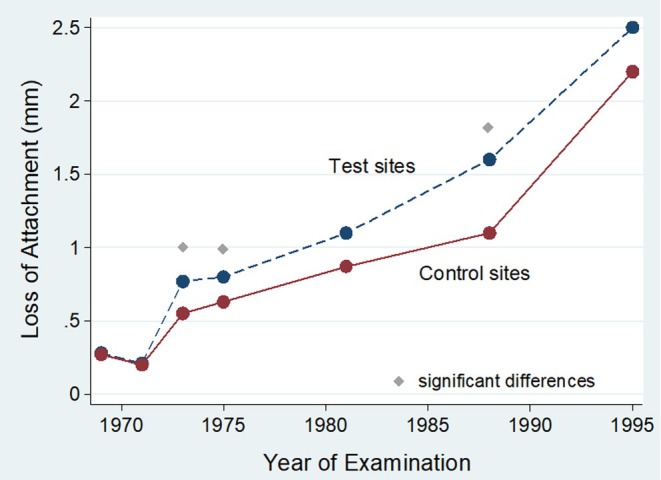
Course of mean attachment loss (mm) for control (*N* = 615) and test sites (*N* = 98) sites in 160 participants over 26 years. At each of 7 examinations, the mesial and buccal sites of premolars and molars were scored for dental, restorative and periodontal variables. Control group had sound surfaces or filling margins located more than 1 mm from the gingival margin in all 7 surveys, whereas test sites were filled within the first 2 years of observation and had subgingival filling margin. Restorations placed below the gingival margin were detrimental to periodontal health, but deterioration plateaued after 3 years. The data was collected between 1969 and 1995 in Oslo, Norway.^77^

**FIGURE 8:**
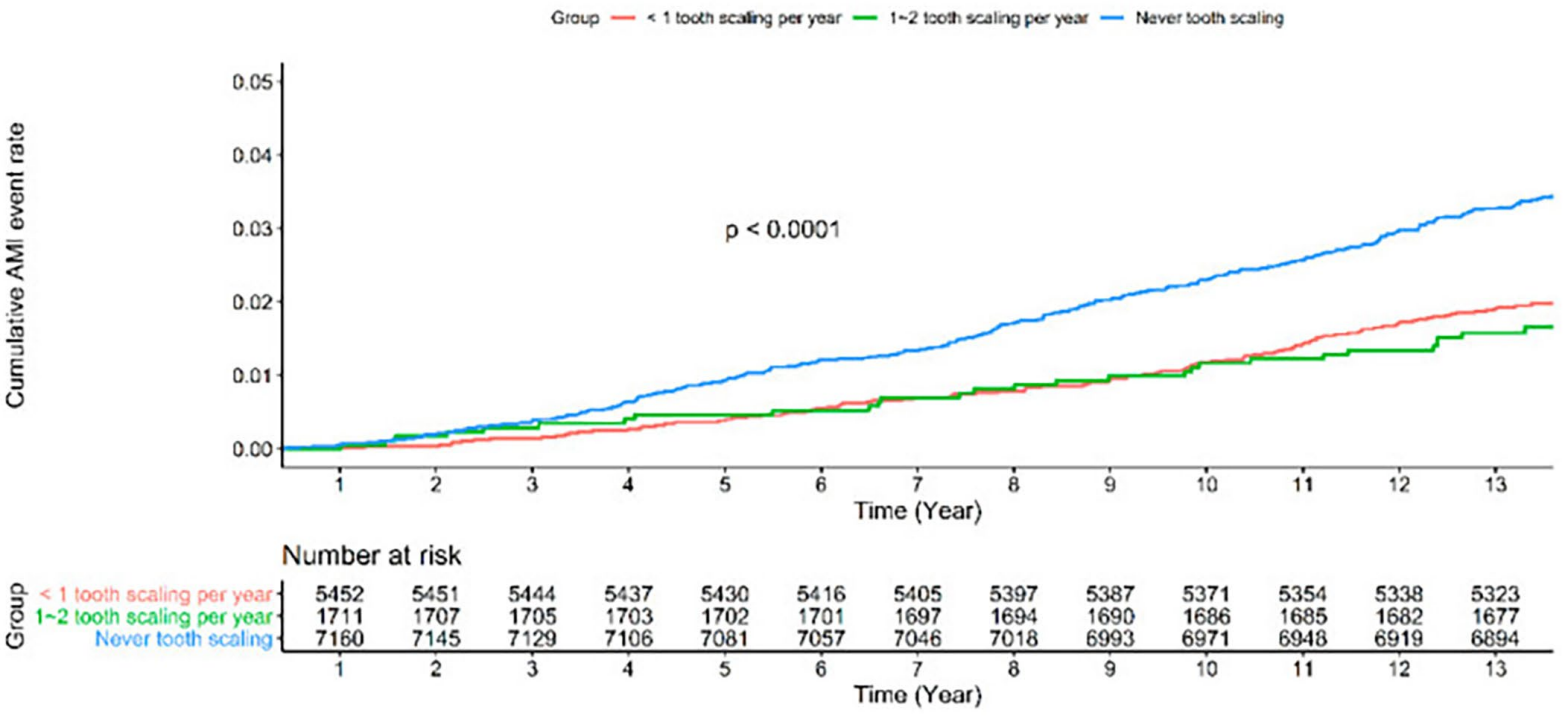
In total, 7164 participants who underwent tooth scaling were compared to 7164 participants without tooth scaling through propensity score matching to assess AMI risk by Cox's proportional hazard regression. Participants with scaling had a lower cardiovascular risk than subjects without scaling. The data source was the Longitudinal Health Insurance Database (LHID), which was provided by the Taiwan National Health Research Institute.^78^

**FIGURE 9:**
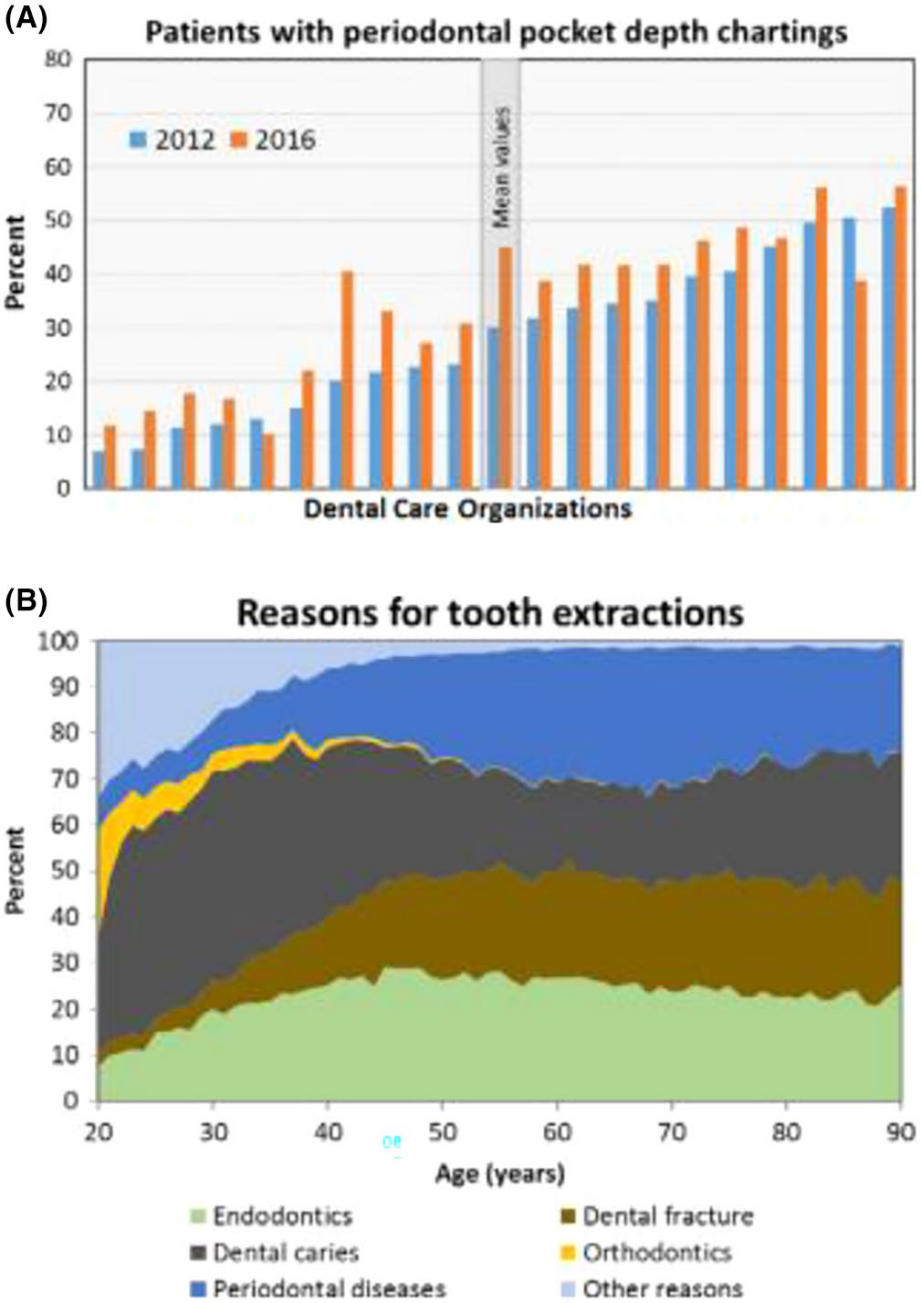
(A) Percentage of patients (≥20 years) with documented periodontal pocket depth in the various dental care organizations (Number of patients: 1 288 462 in 2012 and 1 307 573 in 2016). Although periodontal pockets were assessed, it seems without consequence. (B) Distribution of reasons for tooth extractions in 2016 in 20‐90‐year‐old patients: endodontics, dental caries, periodontal diseases, dental fracture, orthodontics, and other reasons. Number of patients in 2016 was 1 337 145; number of patients with one or more teeth extracted was 91 468; total number of extractions was 207339.^79^ The data is taken from the national Swedish Quality Registry for Caries and Periodontal Diseases (SKaPa), which was launched in 2008 and covers treatment carried out by around 8800 dentists and 4000 dental hygienists.

**FIGURE 10:**
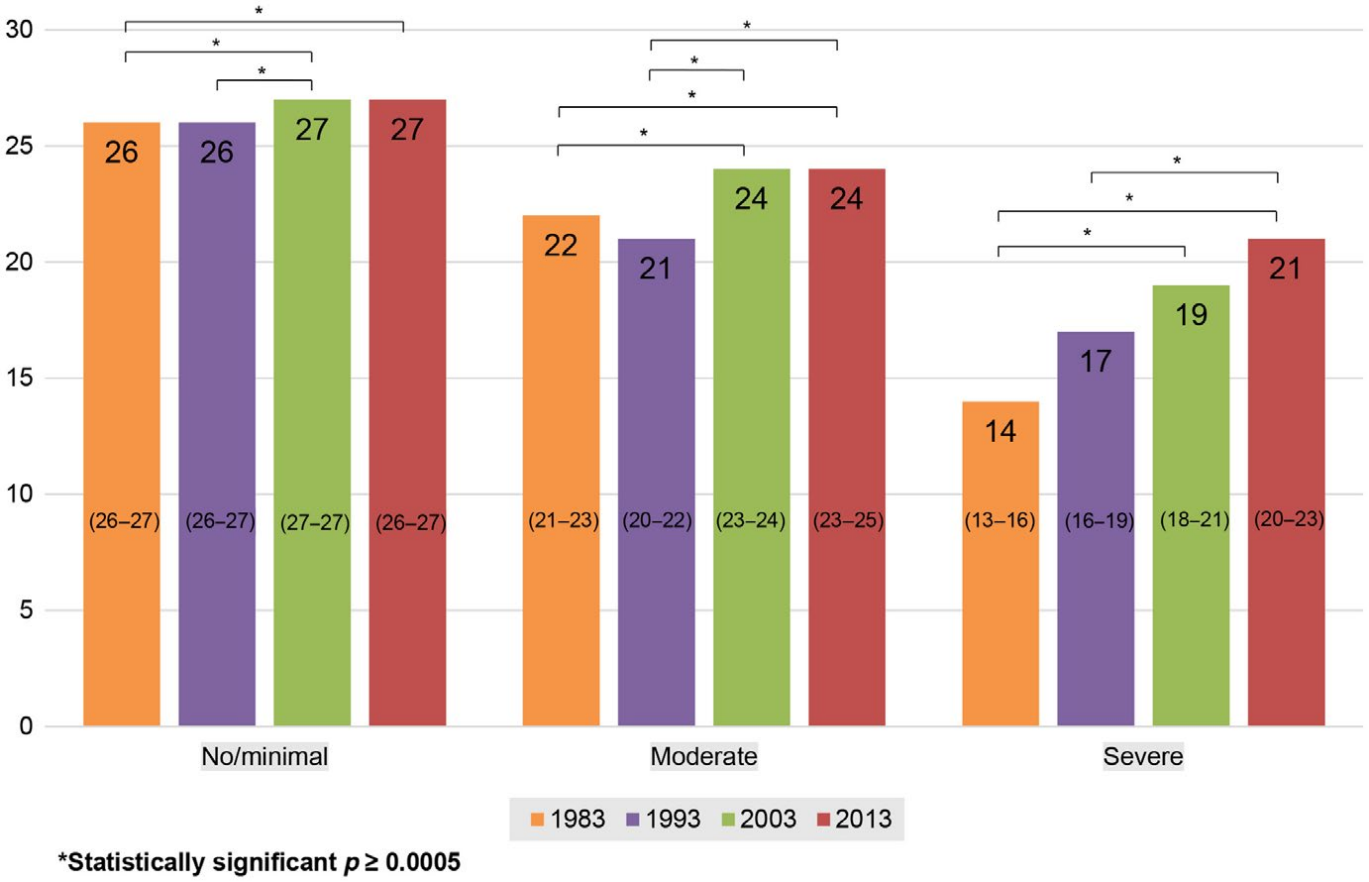
Mean number of teeth (with 95% confidence interval; in dentates) in the age groups 20–80 years between 1973 and 2013. Representative cross‐sectional examinations have been repeatedly conducted every 10 years in the city of Jönköping, Sweden. The number of teeth increased significantly between 1983 and 2013. In 2013, the number of teeth was markedly higher in the severe periodontitis group.^80^

**FIGURE 11:**
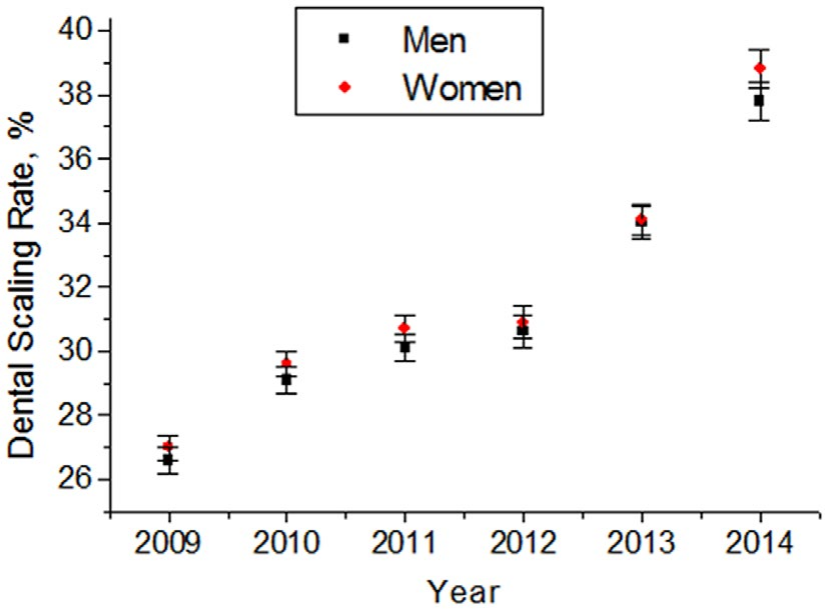
Temporal change in dental scaling rates and differences for men and women. Scaling rates rose about 30% from 2012 to 2014, because remuneration was in part paid by the insurance. Secondary data for 3 175 584 participants from the Community Health Survey (2009–2014) in Korea.^81^

**FIGURE 12:**
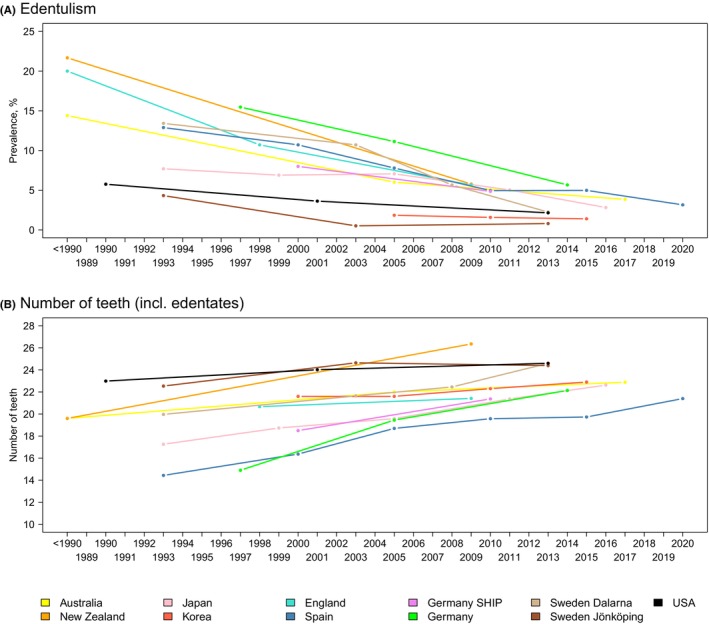
(A) Trend in the prevalence of edentulism for each of the population‐based studies across the corresponding time points. In all cohorts, there was a clear trend towards lower prevalences of edentulism. However, the prevalence data can only be compared with each other to a limited extent, as the age composition of the cohorts is different. (B) Trend in the mean number of teeth (including edentates); in almost all cohorts, the mean number of teeth at the last examination was above 20 teeth. However, it should be noted that in Australia, New Zealand, Japan and England the wisdom teeth were included in tooth counts. Data were collected in Australia from the Adult Oral Health Study, in New Zealand from the New Zealand Oral Health Survey, in Japan from the National Survey of Dental Disease, in Korea from the Korea National Health and Nutrition Examination Survey, in England from the Adult Dental Health Survey, in Spain from the Encuesta de Salud Oral en España, in Germany from the Deutsche Mundgesundheitsstudie and from the Studies of Health in Pomerania, in Sweden from the Jönköping Study and the Dalarna studies, in the USA from the National Health and Nutrition Examination Surveys.

**FIGURE 13:**
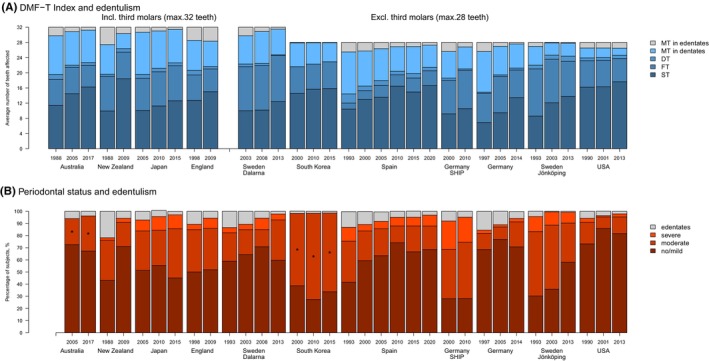
(A) Distribution of DMF‐T components for each population‐based study at the respective study time points. In contrast to conventional plots, in which the DMF‐T index refers to dentate subjects only, we also included edentulous subjects (via MT in edentates). This was done to get an overall picture of the caries situation within the respective population. Although it seems counterintuitive to assign missing teeth to dentate and edentate subjects separately in the DMF‐T Index, we can thereby obtain better conclusions about trends across all populations. Some studies considered 32 teeth as the basis of the DMF‐T (left), while others considered 28 teeth (right). In all cohorts it is clear that the proportion of healthy teeth increased from an average of 10 to 15 during the last three decades and the average number of filled teeth remained almost unchanged, with the exception of Sweden Jönköping Study. In all cohorts, except for England and the USA, the average number of missing teeth in edentate subjects decreased. Compared to the other countries, the average number of filled teeth was lowest in Spain, but the total number of teeth present was comparable to Australia, Japan and both German cohorts. In these countries, the average number of filled teeth was comparably higher. It should be noted, that data from different countries can only be compared to a limited extent, as the age composition of the cohorts is different. (B) Distribution of periodontitis prevalence including the prevalence of edentulism for each of the population‐based studies at the respective study time points. In contrast to conventional plots, in which periodontitis prevalence refers only to dentate subjects, we also included the edentulous subjects to obtain an overall picture of the respective changes within different populations. Periodontal status was categorized as no, moderate or severe periodontitis. For the Australian and the Korean cohort (*), prevalences for moderate and severe periodontitis were reported together. In contrast to the DMF‐T index, the periodontal status did not show any trend within populations; only in the cohorts of New Zealand, Spain, Sweden Jönköping and the USA the proportion of healthy individuals had increased. Data were collected in Australia from the Adult Oral Health Study, in New Zealand from the New Zealand Oral Health Survey, in Japan from the National Survey of Dental Disease, in Korea from the Korea National Health and Nutrition Examination Survey, in England from the Adult Dental Health Survey, in Spain from the Encuesta de Salud Oral en España, in Germany from the Deutsche Mundgesundheitsstudie and from the Studies of Health in Pomerania, in Sweden from the Jönköping Study and the Dalarna studies, in the USA from the National Health and Nutrition Examination Survey.

**FIGURE 14:**
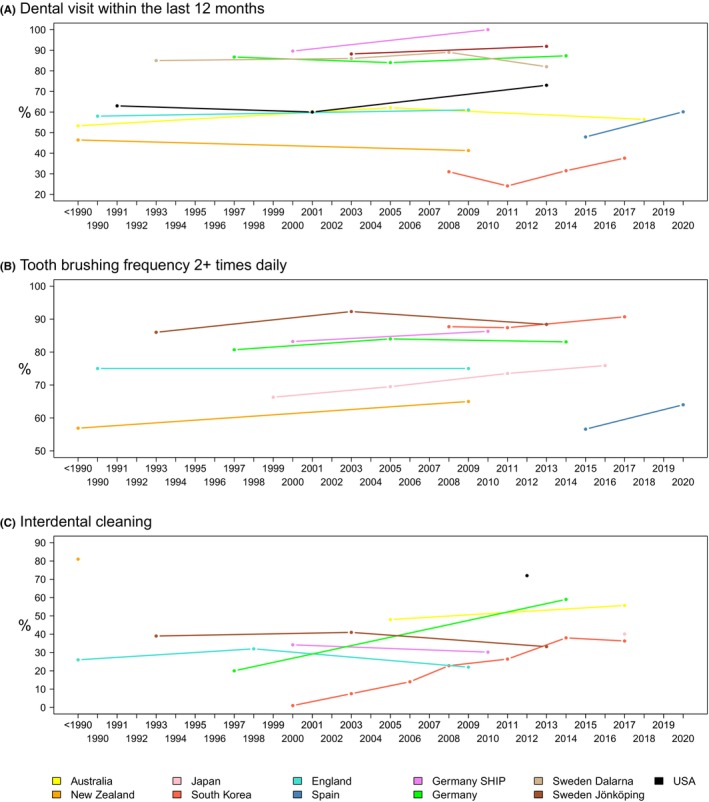
(A) Change in percentage of subjects reporting at least one dental visit in the past 12 months (information available for almost all cohorts). (B) Change in percentage of subjects who reported brushing their teeth at least twice a day (information available for all cohorts). Overall, there was little change in tooth brushing frequencies. (C) The proportion of subjects reporting interdental cleaning increased in three out of six cohorts. Data were collected in Australia from the Adult Oral Health Study, in New Zealand from the New Zealand Oral Health Survey, in Japan from the National Survey of Dental Disease, in Korea from the Korea National Health and Nutrition Examination Survey, in England from the Adult Dental Health Survey, in Spain from the Encuesta de Salud Oral en España, in Germany from the Deutsche Mundgesundheitsstudie and from the Studies of Health in Pomerania, in Sweden from the Jönköping Study and the Dalarna studies, in the USA from the National Health and Nutrition Examination Survey.

Large systematic reviews have shown that social inequality is significantly associated with caries experience, irrespective of whether social inequality was defined as education, income, or occupation.^82^ Interestingly, the association between lower levels of education and higher caries experience was more pronounced in highly developed countries than in underdeveloped countries.^83^

### 4.2 Smoking control

As early as 1952, Doll et al. identified the causal link between smoking and lung cancer.^84^ Nevertheless, prevalence of smoking and tobacco‐related diseases continued to rise in the affluent world. It took about two decades for the public and health policy makers to realize that tobacco consumption could only be reduced through various approaches such as health warnings on tobacco packages, marketing restrictions, regulatory measures, etc. Since the mid‐1970s tobacco consumption among adults in affluent countries has steadily declined as a result of a variety of regulatory measures.^85^ For example, 20 years ago, smoking was very common in bars or on trains or planes in separate compartments, whereas today it is banned from these places. Data from the Organization for Economic Cooperation and Development (OECD) clearly showed that for the selected high‐income countries, smoking prevalence had steadily decreased from 20%–35% in 1995 to 8%–16% in 2020 (Figure 2B).

Many studies around the world have confirmed the early 1980s findings that tobacco smoking is detrimental to periodontitis and tooth loss.^76^, ^86^ Between 50% and 60% of the periodontitis burden of the US population can be attributed to smoking according to data from the National Health and Nutrition Examination Survey (NHANES III).^54^, ^87^ Based on the Australian National Survey of Adult Oral Health 2004–2006, the population‐attributable fraction of smoking was 32% for moderate and 56% for severe periodontitis. If no one in the population had smoked, more than 50% of severe cases could have been prevented.^88^ For periodontitis the smoking attributable fraction appears to be around 50% in all countries. It should be remembered, however, that these rates are based on the unrealistic assumption that no one smokes in a given population.

There are only few studies, that examined the relationship between changes in smoking rates and changes in the prevalence or incidence of periodontitis at a population level. One of them showed that the imputed incidence for advanced periodontitis in the USA decreased 31% between 1955 and 2000 due to a continuous decline in smoking.^10^ Based on national smoking rates from Swedish National Statistics, Bergstrom calculated periodontal disease prevalence using the concept of population‐attributable fraction between 1970 and 2010.^76^ The age‐standardized smoking rate decreased from 44% in 1970 to 15% in 2010 (Figure 5). In parallel, the estimated prevalence of periodontitis decreased from 23.0% to 13.4% assuming a 5‐fold smoking‐associated relative risk. Without smoking, about 65% of periodontitis cases would not have occurred in 1970 and 40% in 2010. Furthermore, that periodontitis prevalence would further decrease to 10.0% if the smoking rate was reduced to 5% in 2030. A Japanese 10‐year longitudinal study with 1466 participants extended such an analysis to tooth loss and calculated a partial population percentage attributable risk for current smoking of 13.4%.^89^ A multivariate analysis of repeated cross‐sectional data from three population‐based studies (DMS, SHIP, Jönköping) showed that decreasing trends in current smoking lead to more present teeth.^67^ Between DMS III and DMS V, the average number of teeth in adults (seniors) increased from 24.0 to 26.2 (14.5 to 19.4), while the proportion of current smokers decreased by 9%. This decrease in the proportion of current smokers accounted for 0.07 more teeth. Similar trends, although not statistically significant, were observed in the SHIP and the Jönköping studies.

Taken together, smoking is the strongest modifiable driver of periodontitis and a major risk factor to be targeted, although the decline in smoking is much more pronounced in people with higher education than in those with lower education.^10^ Smoking reduction is highly dependent on legislation in each country. But besides the legislation, oral health care teams have their place to advise individuals to stop smoking.^86^

While smoking is a well‐established risk factor for periodontitis, the effect of smoking on dental caries has been less frequently studied. Smoking appears to increase dental caries risk, but more well‐designed prospective studies are needed to confirm these findings.^86^, ^90^, ^91^

### 4.3 Diabetes

The prevalence of type 2 diabetes mellitus (T2DM) has been increasing in high‐income countries for decades (Figure 2C). Drivers for T2DM are likely to be sedentary lifestyles and dietary changes, characterized by high consumption of animal products, refined grains, hidden sugars and sugar‐sweetened beverages. A landmark cardiological study (PREDIMED) showed the positive impact of healthy diets on reducing CVD events and mortality.^92^ As with many chronic non‐communicable diseases, diet also plays a critical role in etiology and prevention of periodontal disease. In addition to biofilm as a necessary cause, the host response, which is also related to diet, has received more attention in the periodontal field over the past years. In 2009 a small Swiss study showed that a “Stone age diet”, which is rich in fibers, fish oils, and micronutrients, reduced bleeding and pocket depth compared to baseline.^93^ This study sparked a lot of interest in the interaction between diet and periodontitis, but reliable evidence of the influence of diet on periodontitis is still lacking.

However, the effect of an unhealthy diet, which is well reflected by the effect of T2DM on periodontitis, is well documented. Patients with diabetes more often suffer from periodontitis compared to people without diabetes. Two recent meta‐analyses compared the relative risks of subjects with and without diabetes and reported that diabetes increased the risk of periodontitis progression by 86% (Relative Risk 1.86, 95% CI: 1.3–1.8)^94^ and 24% (Relative Risk 1.24, 95% CI: 1.13–1.37).^95^ Contrary to general opinion regarding the role of diabetes as a risk factor of periodontitis, multivariate analysis of repeated cross‐sectional data from three population‐based studies (DMS, SHIP, Jönköping) consistently showed that the increase in diabetes prevalence was not associated with the presence of fewer teeth. In DMS seniors, the prevalence of diabetes increased from 12.7% to 15.3% between 2007 and 2014, and from 8.2% to 9.6% in the SHIP studies. This null finding may be explained by the increasing proportion of participants with a well‐controlled diabetes and the decrease in unknown diabetes in Germany^96^ (Figure 2D). In SHIP participants, the improved metabolic control is reflected by a decrease in the average HbA1c from 5.4 ± 0.9% to 5.3 ± 0.8%.^67^ Consistent with these observations, well‐controlled diabetes did not increase the risk of periodontitis and tooth loss.^97^, ^98^ Although the prevalence of diabetes in the adult US population increased from 9.8% (95% CI: 8.6–11.1) to 14.3% (95% CI: 12.9–15.8) over the last 15 years, the percentage of undiagnosed individuals decreased from 30% to 23%; the percentage of diabetics with good metabolic control increased from 58.9% (95% CI: 54.4–63.3) to 66.8% (95% CI: 63.2–70.8).^99^ In Sweden, mortality and incidence of cardiovascular events in persons with T2DM decreased substantially between 1998 and 2014; deaths from cardiovascular disease decreased by 110 cases per 10 000 person‐years (95% CI: 91‐129).^100^ In the high‐income countries selected for this review (Australia, Spain, Germany, Japan, South Korea, New Zealand, Sweden, England, USA), the prevalence of diabetes increased from 4% to 8% in 2000 to 6.3% to 13.8% in 2020 (Figure 2C), but the percentage of uncontrolled diabetes clearly decreased in 6 out of 8 countries (Figure 2D). These data are consistent with the observation, that although diabetes increased worldwide, improved diabetes care and improved patient education resulted in reduced rates of cardiovascular complications among patients with diabetes. These observed changes may have simultaneously contributed to the fact that the increased diabetes prevalence did not negatively affect tooth loss.

The effect of diabetes and poor glycemic control on caries has been the subject of several recent systematic reviews.^86^, ^101^, ^102^ Diabetes, in particular poor glycemic control, increases the risk of caries in people with diabetes.

### 4.4 Conclusion

Education is an upstream cause of health and probably one of the best and most cost‐effective investments in public health.^60^ People with higher levels of education tend to have better health than people with lower levels of education because they take preventive health measures, tend to have higher incomes, and have better access to health information and health care.^60^, ^103^ The most important step in promoting health equity is to reduce the education gap between low‐ and high‐income populations. It remains to be seen whether there is a ceiling effect with increasing educational attainment in a given population on the prevalence of periodontitis.

The number of smokers has steadily declined over the past three decades, but tobacco control efforts must continue in the face of tobacco industry lobbying. Smoking cessation in a population depends on the activities of regulatory bodies, but also on social norms. The tobacco industry promotes smoke‐free innovations as less disease‐causing alternatives to cigarette smoking, although this is not proven. It remains to be seen to what extent e‐cigarettes will counteract the effects of smoking cessation on periodontitis. In terms of oral health, the more smoking is reduced, the more oral health improves.

The extent to which the diabetes epidemic is counteracting improvements in oral health cannot be assessed at this time. Over the last 30 years, the increased production of processed foods has led to a shift in unfavorable dietary patterns. If diets could be changed from less sugar, sodium, and saturated fats to more vegetables, fruits, whole grains, legumes, etc., the diabetes epidemic would likely be attenuated. To implement a shift in dietary patterns public regulations will be necessary. The extent to which oral health will be affected is debatable.^104^

## 5 EFFECTIVENESS OF CONSUMER PRODUCTS FOR ORAL HEALTH

Very few long‐term RCTs have evaluated the effects of mouthwash or interdental brushing on attachment loss, caries or on tooth loss. Because progression of periodontitis is slow and tooth loss is a rare event, long‐term trials with attachment loss or tooth loss as endpoints would require large numbers of participants and would be very expensive, unfeasible to perform and probably unethical. Instead, observational epidemiological studies can provide important insights. In Germany, the United Kingdom, the USA, Japan, and South Korea, average sales per capita on oral care products (toothpaste, mouth rinses, dental floss, and manual toothbrushes) were between 10.19 and 19.76€ in 2014 and between 13.37 and 23.75€ in 2020.^105^ Sales volumes of battery and rechargeable power brush handles have markedly increased in Japan, Germany, and the US between 2001/02 and 2020/21 (Figure 6).^106^ So the question is whether consumers are getting better oral health in return for spending more on oral health products.

### 5.1 Mouthwashes

Mouthwashes have a proven effect on gingivitis in short‐term studies.^107^ They are recommended to be used as an adjunct to mechanical toothbrushing. In Australia, about 60% of subjects rinsed their teeth in the past seven days.^108^ In Puerto Rico, 43% of participants used mouthwash at least once a day and in a follow‐up assessment 3 years later, mouthwash use increased to 59% and never‐use decreased from 47% to 26%.^109^ In Scotland, 25% of adults used mouthwash daily, while 38% never used it.^110^ In the USA, 33% of adults rinsed daily and 38% never.^48^ The volume of mouthwashes sold remained virtually unchanged between 2001 and 2022.^106^ In Germany, its use increased from 25.3% to 37.0% in adults and from 39.0% to 41.5% in the elderly between 1997 and 2014.^111^ None of the cited studies evaluated the brand of mouthwash and its potential use in preventing gingivitis or caries. In summary, a very substantial portion of the population uses mouthwashes.

#### 5.1.1 Randomized controlled studies

In a network meta‐analysis of 21 RCTs essential oils showed greater efficacy than triclosan‐copolymer, chlorhexidine, and cetylpyridinium chloride on gingival inflammation in 6‐month home use trials.^107^

#### 5.1.2 Observational studies

Despite widespread use, the effectiveness of mouthwashes in the general population is rarely reported. A study from Puerto Rico reported no notable difference in bleeding on probing (BOP) between subjects who rinsed <2 times (*N* = 738) and those who rinsed ≥2 times daily (*N* = 207).^109^ Both groups had a BOP score of approximately 12%. In an Australian cross‐sectional study of 4170 dentate participants, mouth rinse use was not associated with plaque and attachment loss, but with less gingivitis and lower pocket probing depths (PPD). The authors argued that these contradictory results were probably due to reverse causality.^108^ Their arguments are consistent with a Scottish cross‐sectional study (*N* = 3022), in which participants, who were aware of periodontal problems, rinsed more often than those, who were not aware of periodontitis.^110^ Using repeated cross‐sectional data of the German national oral health studies (DMS III and DMS V; 17 years apart), we investigated, whether more frequent use of mouth rinses was reflected in a higher number of present teeth.^67^ Adults in DMS V had 2.26 more teeth than their counterparts in DMS III, but the 12% increase in mouthwash use in adults did not explain the higher number of present teeth (Figure 3). In contrast to these findings, gargling with a mouth rinse was significantly associated with a lower prevalence of periodontitis among elderly Koreans (Odds Ratio (OR) 0.39; 95% CI: 0.21–0.74).^112^ Well‐designed and long‐term prospective studies with sufficient power are needed to evaluate whether mouth rinses are effective in promoting oral health and whether they offer good value for money for patients.

### 5.2 Powered toothbrushes

Population‐based data on the use of powered toothbrushes are scarce in the dental literature. In East Germany 18% of participants used a powered toothbrush in 2002 and 37% 11 years later.^113^ Nationally, powered toothbrush use increased from 14% to 47% in Germany between 1997 and 2014,^111^ from 2% to 10% in Korea between 2000 and 2006,^114^ and to about 20% to 25% in 2012/14,^115^ from 6% to 27% in the United Kingdom between 1989 and 2008,^35^ and from 30% to 38% in Spain between 2015 and 2020, according to phone interview.^116^

#### 5.2.1 Randomized controlled trial

Recent metanalyses of RCTs concluded, that powered toothbrushes removed plaque more efficiently than a manual brushes^117^ and were more effective in reducing gingivitis and bleeding scores.^118^ The differences were moderate, with powered toothbrushes reducing 21% more plaque and 11% more gingivitis than manual brushes after three months of use. Whether these modest improvements also translate into a long‐term clinical benefit, remained unclear.^119^ Four clinical RCTs in periodontally healthy patients or patients undergoing periodontal maintenance provided an answer to this question. Three of these trials had a follow‐up of 3 years with up to 160 participants. In two trials, the test group used a powered brush together with a Triclosan‐containing toothpaste; the control group used a manual brush with a standard toothpaste. In the three trials with a 3‐year follow‐up, the two groups did not differ significantly in PPD or clincial attachment levels (CAL) at the final examination.^120^, ^121^, ^122^ However, in a 6‐month study with maintenance patients a small advantage was found for powered toothbrush users over manual toothbrushes at the final examination: mean PPD was reduced by 0.15 mm and 0.2 mm in manual and powered toothbrush users (*p* = 0.07), respectively; mean CAL was reduced by 0.06 mm and 0.17 mm in manual and powered toothbrush users, respectively (*p* < 0.05).^123^

#### 5.2.2 Observational studies

In a cross‐sectional study with a convenience sample of 2016 subjects, powered tooth brush users had a small advantage over manual brushers in terms of gingival bleeding in an unadjusted analysis.^124^ Consistent with short‐term RCTs, but in contrast to mid‐term RCTs, long‐term observational studies have provided evidence for the beneficial use of powered toothbrushes. In a 10‐year retrospective study (*N* = 201), patients in periodontal maintenance cleaned their teeth either with a manual or a powered, rotary brush. Twenty‐five percent of the manual and 67% of the powered brushers had good to very good oral hygiene during maintenance and powered brushers lost 0.082 teeth/year compared to 0.185 teeth/year for manual brushers (crude unadjusted values).^125^ In a longitudinal study with an 11‐year follow‐up (SHIP‐START‐1 to −3), the effects of powered toothbrush use on periodontitis, caries, and tooth loss in an adult population were evaluated using multivariate analyses (*N* = 2819). Powered toothbrush users had 0.19 mm less mean attachment loss, 18% less DMFS progression and retained 20% more teeth than manual brushers.^113^ Using 7‐year follow‐up data of 2214 SHIP‐TREND‐0/−1 participants, powered brushers had lower mean PPD (1.73 vs 2.21 mm) and mean CAL than manual brushers (1.96 vs 2.30 mm).^126^ An adjusted analysis of the 6th Korean National Health and Nutrition Examination Survey (KNHANES) wave (*N* = 18 382 participants) found a lower risk (OR 0.77, 95% CI: 0.61–0.97) of having CPI scores of 3 and 4, when using a powered toothbrush.^115^ In an Oaxaca decomposition analysis based on two repeated cross‐sectional DMS studies with a 17‐year time lag, the more frequent use of powered toothbrushes explained only a minute difference in PPD both in adults and seniors, but 1.72 out of 46.3 additional caries‐free healthy surfaces and 0.49 out 4.49 additional teeth in seniors (Table 2).^111^ These long‐term data from two large population‐based studies suggest that the use of a powered brush is beneficial for long‐term oral health.

**TABLE 2:** Changes in the use of power toothbrushes and interdental cleaning aids explained changes in prevalence of periodontitis, caries, and tooth loss over the course of 17 years, using DMS III‐V data. Blinder‐Oaxaca Decomposition was applied.

Outcome	Predicted Means					
	DMS III	DMS V	Total change	Total change explained	Of Which: Change explained by PTB	Of Which: Change explained by IDA
Adults (35–44 years)						
Mean PPD, mm	2.12	2.20	+0.08	−0.10*	−0.01	−0.04*
Healthy surfaces	69.94	108.35	+38.41	+2.58*	+0.64	+0.58
Number of teeth	24.17	26.29	+2.12	+0.65*	+0.11*	+0.22*
Seniors (65–74 years)						
Mean PD, mm	2.49	2.70	+0.22	−0.15*	−0.02	−0.05
Healthy surfaces	35.40	81.69	+46.29	+8.44*	+1.72*	+5.80*
Number of teeth	15.18	19.67	+4.49	+2.19*	+0.49*	+1.25*

Abbreviation: PPD, pocket probing depth. *Source*: Pitchika et al.^111^

* *p* < 0.05.

### 5.3 Interdental aids (dental floss, interdental brushes)

Since the landmark study of experimental gingivitis, oral hygiene has been considered as the cornerstone of maintaining periodontal health.^127^ However, brushing with a toothbrush is not sufficient to clean the interdental spaces. Dental floss, interdental brushes, wooden or plastic picks have been advocated to overcome this problem.

In the United Kingdom, 18% of younger people (18–24 years) and 37% of older people (54+ years) flossed daily.^128^ In the USA, the prevalence of daily flossing has remained at around 30% of the population for the last 40 years.^129^, ^130^, ^131^, ^132^ In Korea, flossing increased from 12.9% in 2007/2009 to 24.0% in 2016/2018 among adults aged ≥30 years.^30^ In Germany, flossing increased from 20.9% to 35.0% in adults and from 4.1% to 11.6% in seniors between 1997 and 2014.^41^ In 2004/06, 20% of Australians cleaned their interdental spaces daily, and another 40% regularly but not daily.^108^ In the USA, interdental brushes are relatively uncommon,^132^ whereas in Europe and Asia, interdental brushes have become increasingly popular over the last 20 years. In Korea, the use of interdental brushes increased from 12.6% (2007/2009) to 18.7% (2016/2018).^30^ In Germany, frequencies increased from 3.5% to 6.7% in adults and from 8.7% to 18% in seniors between 2005 and 2014.^41^, ^111^ These data are not directly comparable because the reported frequency may vary between unspecified and daily use. Nevertheless, these repeated cross‐sectional studies show that interdental cleaning has become more popular over the past two decades, with approximately 20% to 60% of the population reporting flossing and/or interdental brushing.

#### 5.3.1 Randomized controlled trials

Many clinical RCTs have been conducted to evaluate the efficacy of these aids in short‐ and medium‐term studies. As outcomes, the reduction in supragingival plaque and/or gingival inflammation was usually evaluated. A recent Cochrane metanalysis included 35 RCTs with 3929 participants that evaluated the effect of interdental cleaning aids as an adjunct to toothbrushing.^133^ They concluded that adjunctive use of dental floss or interdental brushes may reduce gingivitis or plaque better than tooth brushing alone, while interdental brushes may be more effective than dental floss. However, none of the studies to date have evaluated caries as an outcome in adults.^133^ A scoping review reported weak benefits of these adjunctive devices for plaque and gingivitis control and concluded that long‐term studies with sufficient power are needed to assess the impact of interdental cleaning on oral health.^134^

#### 5.3.2 Observational studies

A cross‐sectional study conducted in Detroit with 319 participants reported that daily flossing reduced plaque and gingivitis scores, but was not associated with reduced probing depths or periodontal attachment loss.^129^ This exceptional and unique study also assessed flossing dexterity, using five criteria (holds floss firmly, eases floss through contact point, pushes floss subgingivally, wraps floss around line angles, moves floss vertically against tooth). These criteria were summed up and based on these criteria, 60% of the 246 flossers had an inadequate technique. Plaque accumulation, but not gingivitis scores differed between good and poor flossers, and mean PD and mean CAL were significantly lower for good flossers than for poor flossers (mean PD 2.21 ± 0.48 mm vs 2.41 ± 0.59 mm, mean CAL 0.96 ± 1.0 mm vs 1.33 ± 1.5 mm). To our knowledge, this is the only epidemiological study, in which flossing skill was judged by face‐to‐face observation. The results support the clinical intuition that many subjects do not floss properly. When done properly, it contributes to better oral health.

In a questionnaire‐based, adjusted analysis of a small subgroup of Health Professionals Follow‐Up Study and Nurses Health Study participants, persons flossing <1 daily were as likely to have periodontitis as daily flossers (OR 1.16, 95% CI: 0.63–2.13).^135^ Unfortunately, the authors did not compare flossers with non‐flossers. Cross‐sectional population‐based data from Australia showed a beneficial effect of daily interdental cleaning on plaque and gingivitis, but not on PPDs and CAL.^108^ In contrast, two recent analyses of NHANES found that interdental cleaning was associated with better periodontal health.^136^, ^137^ Both US studies used the same data set (NHANES 2011–2014) but different outcomes. The 1st study only analyzed the effect of interdental cleaning on periodontitis as defined by the Centers for Disease Control and Prevention (CDC)/American Academy of Periodontology (AAP) case definition.^136^ Interdental cleaning at least 2 to 4 days a week lowered the probability of having periodontitis. The 2nd study carried out more detailed analyses and reported dose‐modeling, suggesting that interdental cleaning 4 to 7 times per week had a better effect than cleaning only 1 to 3 times per week (Table 4).^137^ Subjects, who performed interdental cleaning 4 to 7 times per week had about 9% fewer sites with interdental CAL ≥3 mm, 2% fewer sites with interdental PPD ≥4 mm, 0.36 fewer surfaces with coronal caries, 0.19 fewer surfaces with interproximal caries and about 2.2 more teeth than non‐users. In the 5th KNHANES wave (2010–2012), the use of interdental cleaning aids reduced the risk of having a CPI code of 3 and 4 by about 44%.^112^ Interestingly, younger subjects (19–39 years) benefitted from flossing, whereas older subjects (40+ years) benefitted from the use of interdental brushes. Supporting results were reported from an adjusted analysis of the 6th KNHANES wave (*N* = 18 382 participants): flossers had a 32% lower risk (OR 0.68, 95% CI: 0.59–0.78) of having a CPI score of 3 or 4.^115^ A cross‐sectional analysis of the 6th wave of KNHANES (2013–2015) with 13 525 participants extended the view from periodontitis to caries and reported that non‐flossers had a 1.46 (95% CI: 1.16–1.84) higher risk of approximal caries than flossers, whereas the use of an interdental brush was not associated with less approximal caries.^138^

In contrast to cross‐sectional studies, longitudinal studies can detect changes in the outcome in association to an exposure assessed at baseline and can capture the time course. A large representative US questionnaire‐based study with 48 671 participants analyzed the dose effect of interdental cleaning after one year of follow‐up.^132^ Compared with no interdental cleaning, daily interdental cleaning was prospectively associated with higher odds of excellent self‐rated oral health (OR 1.37; 95% CI: 1.17–1.62) and with lower odds of bleeding gums (OR 0.62; 95% CI: 0.54–0.70). No significant associations were found for any of the other subjective oral health variables (tooth loss, loose teeth, bone loss, gum disease). Although the number of subjects was very high, the information content of this report is subject to many uncertainties, because it relied entirely on self‐reported questions and, above all, the follow‐up of one year was too short to detect progression of periodontitis or tooth loss. A longitudinal US study with 736 male participants (VA Dental Longitudinal Study) examined the effect of any flossing (38% of participants) vs. no flossing (62% of participants) on tooth loss over 26 years (Table 3).^130^ Baseline and long‐term hygiene behaviors (except brushing) were associated with a 60% reduction in tooth loss in consistent flossers in an unadjusted analysis. Similar results, based on adjusted multivariate modeling, have been reported from a US cohort of 375 subjects aged 65+ years, who were followed for 5 years.^139^ Flossers (36% of the subjects) lost 1.16 ± 0.47 teeth while non‐flossers (64%) lost 4.22 ± 0.38 teeth. In detail, non‐flossers lost 42% more molars than flossers. These results are in contrast to a Finnish study that examined 1667 dentate adults (30–82 years) in 2000, 2004, and 2011. Only 12% of participants reported daily interdental cleaning either with floss or an interdental brush. Interdental cleaning was not associated with an 11‐year change in the number of teeth with PD ≥4 mm after adjustment.^140^ The authors argued that flossing was a surrogate for social status and therefore not independently associated with oral health.

**TABLE 3:** Number (%) of individuals losing teeth within 5 years by tooth type and flossing habits using data from 375 seniors aged ≥65 years from the Piedmont 65+ Dental Study.

	Non‐flossers	Flossers	*p* value
All molars retained	147 (61.5)	96 (70.6)	
1 or more molars lost	92 (38.5)	40 (29.4)	0.01
All premolars retained	140 (58.6)	112 (82.4)	
1 or more molars lost	99 (41.4)	24 (17.7)	0.0002
All canines retained	148 (61.9)	125 (91.1)	
1 or more canines lost	91 (38.1)	11 (8.1)	<0.0001
All incisors retained	141 (59.0)	119 (87.5)	
1 or more incisors lost	98 (41.0)	17 (11.5)	<0.0001
Sum of each tooth type	239 (100)	136 (100)	

*Note*: Data are presented as numbers (%). *p* values were taken from Cochran–Mantel–Haenszel tests adjusted for race, sex, diabetes, smoking, education, brushing, and dental utilization. *Source*: Marchesan et al.^139^; Reprinted with permission.

**TABLE 4:** Associations between interdental cleaning behavior and interdental clinical attachment level (iCAL) ≥3 mm, interproximal probing depth (iPD) ≥4 mm, coronal and interproximal caries, number of missing teeth. Data were taken from NHANES (2011–2012 and 2013–2014). Source: Marchesan et al.^130^; Reprinted with permission.

Interdental Cleaning Behavior	iCAL ≥3 mm, % sites	iPD ≥4 mm, % sites	Coronal Caries, *n*	Interproximal Caries, *n*	No. of missing teeth, *n*
**A. Non‐users**	30.6 (0.53)	5.77 (0.24)	1.12 (0.04)	0.62 (0.03)	9.53 (0.13)
**B. Interdental cleaning users (1 to 3 times per week)**	22.8 (0.58)	4.37 (0.26)	0.72 (0.05)	0.37 (0.03)	7.60 (0.14)
**C. Interdental cleaning users (4 to 7 times per week)**	21.3 (0.44)	3.77 (0.20)	0.76 (0.04)	0.43 (0.02)	7.32 (0.10)
*p* values					
Overall	<0.0001	<0.0001	<0.0001	<0.0001	<0.0001
A versus B	<0.0001	<0.0001	<0.0001	<0.0001	<0.0001
A versus C	<0.0001	<0.0001	<0.0001	<0.0001	<0.0001
B versus C	0.04	0.07	0.59	0.14	0.11

*Note*: Data are presented as mean (SE). Data adjusted for race, sex, age, diabetes, smoking, education, and dental utilization. Caries, interproximal caries, and number of missing teeth were also adjusted for sugar consumption. Abbreviations: iCAL, interproximal clinical attachment level; iPD, interproximal probing depth. *Source*: Marchesan et al.^137^; Reprinted with permission.

**TABLE 5:** Yearly change of edentulism (as percentage), DMF‐T components (sound, filled, decayed, missing teeth in dentates, missing teeth in edentates) and periodontitis prevalence (%; no/mild, moderate, severe).

Study	Edentulism	Caries – DMF‐T components					Periodontitis		
		Sound	Filled	Decayed	Missing in dentates	Missing in edentates	No/mild	Moderate	Severe
Australia	0.11	0.17	−0.04	−0.02	−0.04	−0.05	‐	‐	‐
Spain	0.26	0.23	0.09	−0.06	−0.19	−0.07	1.05	−0.53	−0.09
Germany SHIP	0.24	0.14	0.13	0.02	−0.12	−0.12	0.01	0.58	−0.27
Germany	0.33	0.34	−0.02	0.01	−0.23	−0.10	1.04	−0.38	−0.10
Japan	0.28	0.23	0.07	−0.02	−0.19	−0.07	−0.56	0.74	0.21
South Korea	0.10	‐	0.10	‐	‐	‐	0.53	‐	0.06*
New Zealand	0.32	0.40	−0.10	0.02	−0.15	−0.14	1.27	‐	‐
Sweden Dalarna	0.24	0.24	0.05	−0.05	−0.03	−0.18	0.04	0.49	0.03
Sweden Jönköping	0.12	0.26	−0.16	0.02	−0.08	−0.04	1.39	−1.04	−0.18
England	0.09	0.20	−0.06	−0.05	−0.11	0.02	0.17	−0.08	0.37
USA	0.03	0.06	−0.04	0.00	−0.03	0.00	0.37	−0.17	−0.04

* Moderate plus severe periodontitis.

An application of the Oaxaca Decomposition Analysis to the DMS III to DMS V data evaluated the extent to which changes in the use of interdental cleaning aids explained changes in the severity of periodontitis, the number of caries‐free surfaces and the number of teeth in adults and seniors. Over 17 years, the use of interdental cleaning aids increased by 32.5% in adults and by 41.4% in seniors. The increased use of interdental cleaning aids explained 0.22 teeth of the additionally observed 2.21 teeth in adults, as well as 5.80 out of 8.44 additionally observed caries‐free surfaces and 1.25 out of additionally observed 2.19 teeth in seniors (Table 2). Participants, who used both dental floss and interdental brushes had the highest benefit in terms of more teeth present.^111^

### 5.4 Conclusion

No definitive conclusion can be drawn regarding the impact of mouth rinses on periodontal disease in the general population. Probably, the question of “mouth rinse usage” in a questionnaire needs to be more specific, because different brands are differently effective.^107^ Furthermore, the benefit of mouth rinses needs to be assessed together with tooth brushing. Therefore, the comparison of data on the use of mouth rinses in different countries is limited. Industry advertising has made the use of mouth rinses very popular‐ between 30 and 60% of the adult population use mouth rinses‐ but the long‐term benefit of regular mouth rinse usage is unknown. Observational cohort studies and repeated cross‐sectional studies are urgently needed to answer this question.

The contradictory results of medium‐term RCTs and cohort studies regarding effects of powered tooth brush use can be explained by the fact that in RCTs both intervention groups performed optimal personal hygiene due to regular maintenance visits with repeated motivation, instruction and prophylaxis, which probably counterbalanced the modest effect of powered tooth brushing, whereas participants of epidemiological studies did not receive intensive instruction and prophylaxis. The conclusion that powered tooth brushing might have beneficial long‐term effects on periodontal health is based on analyses of cross‐sectional KNHANES data, repeated cross‐sectional DMS studies and longitudinal SHIP data. As powered toothbrushes are widely used in the population, and the frequency of use does not seem to be stagnating, further observational studies should estimate their effect on periodontitis, caries, and tooth loss.

A more robust conclusion can be drawn for interdental cleaning aids. In line with and extending the conclusion of a metanalysis based on short‐term studies, that interdental cleaning reduces interdental plaque and interdental inflammation to some extent,^133^, ^134^ the reviewed observational studies support their conclusion and extend this conclusion to periodontitis, caries, and tooth loss. While some clinicians believe that effective flossing is only tenable for highly motivated patients, even the “average” motivated flosser with imperfect dexterity achieves some improvement in periodontal status compared to non‐flossers.^129^ Nevertheless, it would be desirable to increase the proportion of daily flossers beyond 40%. This is doubtful when looking at data from the USA, where the upper limit was around 30%–40%, even though flossing has long been embedded in American hygiene culture.^132^, ^136^ Over the past two decades, the oral consumer industry has introduced interdental brushes as an alternative to dental floss with great success. In Korea and Germany, their use has increased by up to 30% among the elderly. Supporting the recommendations of Amarasena et al.,^134^ dentists should advocate interdental brushes, if patients have wide embrasures, otherwise flossing.^112^

Our findings challenge the statement “No evidence on the effectiveness of floss in adults or under real‐world clinical conditions could be identified” of a recent metanalysis about the preventive effect of flossing on caries.^141^ In our view, unsupervised flossing is also effective in reducing caries^138^ and tooth loss under real‐world conditions.^111^ Analyzing of 5‐year follow‐up data showed that the average tooth loss was about 1 tooth among flossers, whereas it was 4 teeth among non‐flossers.^139^ Even if daily flossing does not remove the complete interdental biofilm, it perturbates the biofilm build‐up, impedes colonization with acid‐producing microorganisms^142^ and reduces the caries experience. In a recent Norwegian study of 484 participants, regular flossing was associated with reduced microbial alpha diversity, probably reflecting gingival health.^142^, ^143^ These data suggest that the higher tooth retention rates may be not only due to less caries, but also to better periodontal health.^67^, ^137^, ^139^ Thus, interdental cleaning may contributes to a lower caries and periodontal disease burden, which, in turn, leads to less tooth loss in the long term.

Irrespective of the country, up to 60% of the population perform interdental cleaning with varying degrees of thoroughness, but there is a clear SES gap.^30^, ^110^ A higher SES is associated with a healthier lifestyle, which in turn also influences an individual's oral hygiene habits.^130^ In KNHANES the lowest income group of non‐interdental brushers had an OR of 1.28 (95% CI: 1.06–1.53) for periodontitis compared to the highest income group. Interestingly, among interdental brush users a low income did not increase the risk of periodontitis. The authors concluded that the use of interdental brushes could reduce inequalities in periodontal health.^144^ We do not know whether industry advertising, cultural norms or individual dentist‐patient relationships have more impact in promoting better oral care, especially in people from lower SES classes.

## 6 EFFECTIVENESS OF DENTAL INTERVENTIONS

### 6.1 Reduced number of filled tooth surfaces

Caries has significantly been reduced in both children and adults over the last decades due to the introduction of fluoridated toothpastes^43^ and in some countries due to water fluoridation.^145^ Reduced caries burden is reflected by more sound tooth surfaces, which, in turn, are associated with fewer subgingival restorations; subgingival restorations retain more plaque, push the plaque front apically and thus promote gingival inflammation. In all the cross‐sectional repeated studies cited, the number of teeth increased, driven by an increase in the number of sound teeth and a decrease in the number of filled teeth.

In a retrospective study of 922 patients, the mean PPD difference between restored and healthy surfaces was between 0.42 mm and 0.66 mm.^146^ Using data from the 7th KHNANES wave (2016–2018) with 12 689 participants (age ≥ 19 years), multivariate regression analyses revealed that the odds of having periodontitis, defined as a CPI score of 3 or 4, was 1.24 (95% CI: 1.05–1.46) for subjects with 6–10 crowns and 1.28 (95% CI: 1.01–1.62) for subjects with ≥11 crowns compared to subjects with no crown as reference.^112^ The detrimental effect of fillings on the periodontal health has been demonstrated not only in cross‐sectional but also in longitudinal studies. In the Dunedin Multidisciplinary Health and Development Study (subjects were born in 1972/1973) the approximal tooth surfaces of 884 participants were assessed for restorations, caries and attachment loss at 26 and 32 years of age.^147^ If an approximal surface had caries or was filled (either before the age 26 or between ages of 26 and 32), attachment loss was about twice as likely to be ≥3 mm compared to a sound site. In a 26‐year longitudinal Norwegian study, surfaces were scored for restorative and periodontal variables.^77^ The control group consisted of 615 sound surfaces or filling margins located more than 1 mm away from the gingival margin during the entire 26‐year observation period. The test group consisted of 98 surfaces with a subgingival filling margin. In teeth with subgingival restorations, attachment was lost 1 to 3 years after the placement of the restorations and then a subsequent “burn‐out” effect was observed (Figure 7).^77^

In a cross‐sectional study conducted in 2005, 16 198 interdental sites of 626 young Swiss Army recruits were radiographically evaluated, out of which 95.8% were sound and 4.2% (682) were filled.^148^ Of these 682 filled sites, 14.1% had overhangs of various sizes. Only overhangs ≥0.45 mm showed a statistically significant higher mean PPD than sound surfaces (2.46 ± 0.64 mm vs 2.88 ± 0.99 mm).^148^ Compared to a similar study conducted in 1985 by the same group of researchers, the prevalence of fillings fell from 20.0% to 4.2% in 2006, and the marginal fit of the fillings with overhangs was reduced from 33% to 14%.^148^ These data demonstrate the impact of caries decline and potential improvement in the quality of restorative dentistry on periodontal health. To our knowledge, Kuonen and his group were the first and only authors to report this effect at a population level.^148^

At this point, we can only speculate that the improved caries situation has led to improved periodontal status, as there was less caries in affluent societies between the 1970s and 1990s, but we cannot estimate the magnitude of the effect.

### 6.2 Regular professional prophylaxis

Regular professional prophylaxis or supragingival scaling and polishing supports the patient's self‐performed oral home care, of which interdental cleaning is an integral part. The Karlstad studies impressively demonstrated that meticulous, self‐performed oral hygiene and regular professional prophylaxis can prevent the progression of periodontitis and caries and minimize tooth loss over 30 years.^149^ The Karlstad studies boosted the transfer of these procedures as the basis of individualized preventive programs in dental offices around the world. In the United Kingdom, 46% of all treatments consisted of examination and scaling and polishing under the National Health Service.^150^ In the latest German DMS V study, 21.8% of adults and 25.5% of seniors reported having had a professional teeth cleaning at least once a year in the past five years,^41^ which they had to pay for themselves.

#### 6.2.1 Randomized controlled trials

Only two RCTs assessed the effectiveness of scaling and polishing in populations. The largest RCT, that evaluated the effectiveness of routine scaling and polishing in a real‐life scenario, was the iQuad trial in the United Kingdom. It compared 6‐ or 12‐month intervals of routine sub‐ and supragingival scaling and polishing versus none and different types of personalized oral hygiene instruction in 63 dental offices with 1877 adult patients with gingivitis or incipient periodontitis. After three years, there were no statistically significant or clinically important differences in gingival bleeding scores between the three scaling and polishing groups or between personalized or usual oral hygiene instruction groups. Thirty‐eight percent of sites were bleeding regardless of intervention.^151^, ^152^ A recent Cochrane review, that included the iQuad trial and a second trial, concluded that adults without severe periodontitis did not profit from regular scaling and polishing with respect to gingivitis, probing depth and oral health‐related quality of life over two to three years of follow‐up.^150^

#### 6.2.2 Observational studies

Bivariate analyses from the German DMS V study showed that caries experience did not differ between adults (35–44 years) with and without prophylaxis, while seniors with regular prophylaxis had higher scores of decayed, filled or missing teeth than their counterparts without prophylaxis (probably due to reverse causality).^41^ But in both age groups, prophylaxis led to more favorable periodontal conditions (prophylaxis vs no prophylaxis: (i) in adults: PD ≥4 mm 9% vs 14%, CAL ≥3 mm 39% vs 45%; (ii) in seniors: PD ≥4 mm 20% vs 28%, CAL ≥4 mm 76% vs 71%).^41^ Based on unadjusted analyses of 13‐year follow‐up data, risk of tooth loss was reduced by 29% in men who flossed and received consistent prophylaxis as compared to men with unfavorable hygiene habits.^130^ However, as results from both studies were unadjusted, effect estimates were probably biased due to confounding.

Taiwan has a national health registry covering 99% of its 23.74 million population. Regular prophylaxis is included in the health plan of almost all Taiwanese, but no results have been published that would be accessible to the broader dental community.^153^, ^154^ In linking data from the national registry on prophylaxis with data on the incidence of myocardial infarction, it was found that the adjusted HR (0.90, 95% CI: 0.86–0.95) was significantly lower in the dental prophylaxis group (*N* = 344 228) with untreated controls as the reference group (periodontally diseased subjects, *N* = 45 575).^154^ Similar results have been published with stroke or esophageal cancer as the outcome.^155^, ^156^

### 6.3 Subgingival scaling or non‐surgical periodontal therapy

Most patients have moderate periodontitis and they can be treated with scaling or non‐surgical periodontal therapy alone. In Germany, flap surgery is rarely performed in general practices.^157^ Even in 14 US periodontitis specialist practices, non‐surgical therapy accounted for over 97% of all procedures performed.^158^ Therefore, we consider only scaling as an approach with a potential impact on periodontal health in the general population. In SHIP‐TREND, 20.4% of all participants reported having undergone periodontal treatment within the last five years.^159^ In DMS V, 14.2% of adults and 24.5% of seniors reported having undergone periodontal treatment within the last five years.^41^ In Sweden, the effectiveness of non‐surgical periodontal treatment was tested in 59 dental clinics with 95 dental hygienists randomly performing two different subgingival scaling protocols on 615 patients.^160^ The primary outcome was pocket closure at 6 months (PD ≤4 mm). Irrespective of the treatment protocol, approximately 69–72% of all initial pockets were closed. These results are comparable to 74% pocket closure in (academic) efficacy studies.^161^ This Swedish study showed that scaling works under everyday conditions in skilled hands.

#### 6.3.1 Observational studies

Real‐world data on PPDs or CAL and tooth loss as outcomes are very rare. A registry‐based 4‐year follow‐up study of 1021 members of the Kaiser Permanente Dental Care Programme showed that periodontitis patients, who received regular maintenance, lost fewer teeth than patients who dropped out of the maintenance scheme.^158^ Treatment was provided either by a periodontist or by dental hygienists under supervision. Regular non‐surgical therapy compared with no therapy reduced tooth mortality rate by 58% and irregular therapy by 48% during a 3‐year follow‐up period. Analysis of data from the most recent German DMS V study revealed results that contradict the expectation that periodontal treatment would reduce the number of sites with PPD ≥4 mm. Treated and untreated adults had 18.2% and 12.0% sites with PPD ≥4 mm, respectively. In treated and untreated seniors, 27.4% and 25.6% of all sites had P≥4 mm, respectively. These results, however, were unadjusted and may be prone to confounding.^41^

To further elucidate the effectiveness of periodontal treatment in Germany, longitudinal registry data of periodontally treated patients from a major German national health insurance were merged with longitudinal data of periodontally untreated and treated SHIP‐TREND participants.^162^ Both groups lived in north‐eastern Germany. Untreated (moderate/severe) periodontitis was defined as having either ≥2 or ≥4 teeth with pocket depths ≥4 mm at baseline. Among moderately or severely affected participants, annual tooth loss was higher in insured patients (0.35 ± 0.75) compared with untreated SHIP‐TREND controls (moderate periodontitis: 0.19 ± 0.36, severe periodontitis: 0.26 ± 0.42). In line with this, treated SHIP‐TREND participants with moderate/severe periodontitis had higher annual tooth loss rates than untreated SHIP‐TREND controls (moderate periodontitis: 0.26 ± 0.42, severe periodontitis: 0.29 ± 0.45). Thus, periodontal treatment did not contribute to tooth retention.^159^ It should be noted, however, that in SHIP‐TREND the data on periodontal treatments performed within the last 5 years were based on self‐reported information, so periodontal treatment could be confused with prophylaxis.

Based on the Taiwanese registry, many studies reported the effects of periodontal treatment on systemic outcomes. For example, periodontal treatment was reported to reduce the risk of atrial fibrillation,^163^ low birth weight,^164^ Parkinson's disease,^165^ stroke,^155^ heart attacks (Figure 8),^78^, ^166^ chronic kidney disease,^167^ pharyngeal cancer,^168^ and various other cancers.^169^ The limitation of all these studies is that smoking was not included as a confounder and SES was only sometimes adjusted for. If the perio‐medicine hypothesis is correct, then successful periodontal treatment alone will reduce systemic inflammation and influence systemic events. It would be interesting for the periodontal community to know what the effects of periodontal treatment was on both periodontitis and tooth loss in Taiwan. The Swedish Quality Registry for Caries and Periodontal Diseases receives daily information on dental care provided from electronic patient records and tracks the dental healthcare of 6.9 million of the 10 million Swedes.^79^ A comparison of the number of periodontal pocket depth chartings in 2012 and 2016 showed an increase in 2016 (Figure 9A). In parallel, information on oral hygiene instruction was given more frequently (10.4% in 2010/11; 17.5% in 2015/16), while scaling and root planing was performed less often in 2015/16 (74.7% compared to 80.6% in 2010/11). This trend was accompanied by less tooth extractions (8.6% in 2015/16 versus 7.4% in 2010/11). Obviously, diagnostic data very well fit declining trends of periodontitis in Sweden, as reported by the Jönköping and the Dalarna studies (Figure 13).

### 6.4 Treatment philosophy and extraction patterns

Periodontitis is the leading cause of tooth loss in adults worldwide.^170^ People with severe periodontitis are at risk of extensive tooth loss, edentulism, and masticatory dysfunction. Although in the Jönköping studies, the percentage of subjects with severe periodontitis remained virtually unchanged from 1993 to 2013 (15% to 11%), the mean number of teeth present in subjects with severe periodontitis increased from 14 to 21 (Figure 10).^80^ A similar observation was made in the Swedish Dalarna study, where the mean number of teeth in subjects with severe periodontitis increased from 17.3 ± 8.3 to 22.6 ± 6.0 from 2003 to 2013, although the percentage of subjects with severe periodontitis did not notably change during this period (10% to 7%).^46^ This increase in teeth can be interpreted as either that the Swedish dental community has learned that teeth with advanced periodontal destruction can be treated and retained, or that severe periodontitis affected fewer teeth within a dentition, resulting in fewer extractions. The latter argument is supported by a recent analysis examining whether the pattern of extractions in individuals with severe periodontitis has changed over the past decade in eastern Germany.^171^ No significant differences in the pattern of maximum baseline clinical attachment levels of the retained or extracted teeth were observed between SHIP‐START‐0 and SHIP‐TREND‐0. Although the number of teeth in dentates increased from 20.7 to 21.6 between SHIP‐START‐0 and SHIP‐TREND‐0,^42^ the change in extraction patterns did not contribute to a higher tooth retention rate. However, the Swedish SKaPa registry showed that periodontitis accounted only for about 25% of tooth extractions in adults and was far from being the main cause of tooth loss (Figure 9B).^79^

### 6.5 Conclusion

In conclusion, the decline in caries prevalence in western countries has been beneficial for periodontal health, as sound teeth retain less plaque and promote less inflammation than filled teeth with subgingival margins. As the DMF‐T status of 12‐year‐old children in high‐income countries decreased below one, this effect is likely to improve periodontal health later in life, but it is not known how much of the improved periodontal health is due to healthier teeth.

It is unclear why scaling and polishing procedures in the United Kingdom have not led to better periodontal health in primary health care centers.^151^, ^152^ Possible explanations for these disappointing results include limited economic resources, lack of patient compliance, or poor execution of the procedure itself. It would be desirable to have study results for the effects of prophylaxis and scaling on dental outcomes to support and strengthen the findings reported for systemic disease end points using registry data from Korea and Taiwan.

More cluster‐controlled RCTs, as well as longitudinal and repeated cross‐sectional observational studies are needed to test the effectiveness of scale and polish procedure before it can be unanimously recommended at the population level. Registry data from different health insurance schemes would help to understand the impact of scale and polish procedures on tooth loss. As tooth loss is one of the most important patient outcomes, changes in tooth extractions can well be interpreted as quality characteristics to assess whether the introduction of modified insurance schemes resulted in better treatment outcomes.

When performed by skilled hygienists or periodontists, scaling reduces probing depths, attachment loss, and tooth loss, as shown in the US or Sweden. In Korea and Taiwan, scaling also appeared to work at the population level. However, the Korean and Taiwanese data provided only indirect evidence, as scaling as an exposure appeared to reduce the negative effects of periodontitis on systemic diseases, but the long‐term effects on dental variables remain unknown. As for scaling and polishing, more observational data would be helpful to understand the effect size of scaling in general populations. In addition to Taiwan and Korea, Sweden has longitudinal data from registries that could provide information on the impact of periodontal treatment in a specific population group.^172^

## 7 HEALTH POLICY AND TREATMENT PROVIDERS

Periodontal health is also influenced by the organization and delivery of dental care. Dental health plans are included in compulsory, optional, or commercial health insurances in a few countries, but the extent, scope and coverage vary widely between countries.^173^, ^174^ Only in very few countries is periodontal treatment included in dental health insurance, while in most countries it must be paid out of pocket. Accurate information about what is covered and what must be paid out of pocket requires detailed knowledge of each country. An excellent overview for European countries is provided by Winkelmann et al.^174^ Worldwide, the information and observations presented are fragmentary. This chapter discussed the extent to which dental health plans in Taiwan, Korea, and Germany have included periodontal treatment in their mandatory coverage and the extent to which the health plan has influenced the burden of periodontitis and reduced health inequalities.

Taiwan introduced a national health insurance to improve life expectancy and to reduce health inequalities.^175^ The National Health Insurance program covers up to 99% of the Taiwanese population. It includes oral care and provides free dental check‐ups and prophylaxis twice a year to promote periodontal prevention.^153^, ^154^, ^176^ In 1995, scaling was included as the only periodontal intervention, but its effectiveness was unsatisfactory and inconsistent due to lack of patient motivation and compliance with maintenance visits. In 2010, this treatment regimen was replaced by the comprehensive periodontal treatment project (CPTP).^153^ CPTP stresses and underlines oral hygiene instruction, plaque control, and compliance.^176^ A comprehensive analysis compared patients who received CPTP (65 342 patients) with patients who received only scaling (106 740 patients).^176^ After 545 days of follow‐up, the CPTP group had a lower adjusted odds for tooth extraction after periodontal treatment than the scaling only group (OR 0.83; 95% CI: 0.81–0.85). Chen et al. stated that “Based on the report from the Taiwan Dental Association, 173,073 patients completed CPTP in 2019, and the mean PPD reduction was 0.73 mm, whereas in sites with an initial PPD ≥5 mm, the mean PPD reduction was 2.79 mm…”.^153^ A small Taiwanese study evaluated the effectiveness of this program and concluded that enrolled patients had better oral health profiles.^177^ If these data truly reflect treatment performed in the general population and not in an academic setting, then these results are very consistent with what can be achieved in academic studies.^161^ The Taiwanese database currently appears to be one of the most comprehensive ones in the world in terms of oral health. It would be desirable to (i) compare oral health status between the only scaling group and the CPTP group and (ii) oral health before and after the introduction of the CPTP in the National Health Insurance scheme. In terms of general health, a comprehensive analysis reported that life expectancy increased and health inequalities decreased after the introduction of the national health insurance in Taiwan.^175^

In South Korea, health care is universal. In 2000, all health insurance schemes merged into a single National Health Insurance (NHI), which covers a maximum of 60% of each medical bill. Since 2009, the NHI has expanded its coverage to include dental care. To improve access and to reduce inequalities in periodontal disease burden, the Korean government included annual dental scaling in health insurance in 2013, thereby reducing the costs paid out of pocket from $50 to $10 per session.^81^ This policy led to an increase in the use of regular scaling sessions from 27% in 2009 to 38% in 2014. The effectiveness of scaling under the amended health plan was evaluated using the CPITN in KNHANES, comparing data from 2010–2012 with data from 2016–2018. The results were not consistent: the prevalence of periodontally healthy individuals (CPITN 0) increased from 34% to 39%, and the number of people requiring scaling decreased by 5% (from 66% to 61%). Unfortunately, the data are inconclusive, as the authors also reported that the prevalence of periodontitis (CPITN 3 + 4) increased from 23% to 31% after scaling was covered by health insurance. Several studies evaluated the effectiveness of the health insurance coverage of dental scaling (introduced in 2013) on periodontal health, modeling it as an exogenous variable. After introduction of the scaling coverage policy, the odds of healthy periodontal tissues was 1.10 times higher and the odds of periodontal disease was 0.90 times lower compared to the pre‐implementation period.^178^, ^179^ For the periodontal community, an in‐depth analysis of these real‐world data would be very interesting to understand the impact of scaling from a population perspective. In Korean adults, oral health status has improved over the past decade in parallel to increasing dental scaling rates (Figure 11), but the gap in oral health inequalities according to income level has widened.^52^, ^81^, ^180^ A sobering conclusion was reached that the amended health plan has not reduced oral health inequalities, but has in fact unintentionally increased them.

In Germany, motivation, instruction, and maintenance was not reimbursed by the German statutory health insurance until 2021. Scaling of teeth with PPDs ≥4 mm was reimbursed by the statutory health insurance scheme. However, such a periodontal treatment scheme lacked effectiveness in the general population.^159^ The reimbursement scheme was changed in 2021 and now covers instruction and motivation and a short maintenance period after scaling and flap surgery treatment. The future will show whether this new reimbursement scheme will improve the effectiveness of periodontal treatment and lead to more long‐term tooth retention of periodontally damaged teeth.

Regardless of the extent to which oral health is or is not covered by an universal national health insurance, there are income‐ or SES‐related inequalities in access to dental care.^181^, ^182^, ^183^ Despite complete or partial coverage of periodontal treatment, socially disadvantaged individuals did not have better access to periodontal treatment both in Japan^184^ and Korea.^81^, ^179^ However, the Korean example also shows the limitations: the better educated tend to benefit from greater coverage of services, and the expansion of services does not lead to the desired reduction in the health gap.^185^ Future analysis of registry data from Sweden, Taiwan and Korea may show whether periodontal treatment leads to retention of more teeth.

## 8 TRENDS IN ORAL HEALTH IN WESTERN COUNTRIES

In this review, we searched exclusively for repeated cross‐sectional national and regional studies, which were conducted between 1988 and 2020 in high‐income countries and reported information on the prevalence of edentulism, periodontitis, mean number of teeth and mean DMF‐T scores (Table 1). We believe that this complete dental information is necessary to assess changes in oral health over time and to determine the extent to which changes in the prevalence of periodontitis may have contributed to changes in the number of teeth present. In many publications on periodontitis prevalence, prevalence data are limited to dentate subjects, which gives a distorted picture of periodontitis prevalence.^186^ In order to correctly estimate the prevalence of periodontitis in the general population, information on edentulism is required: if it is assumed that 40% of the population is edentulous and 18% of dentate individuals have periodontitis, the actual prevalence of periodontitis in the population is 10.8%.

We extracted data from a national and a regional German study,^41^, ^187^, ^188^, ^189^, ^190^ from two regional Swedish studies,^45^, ^46^, ^47^ and national studies from Spain,^38^, ^191^ England,^34^, ^35^ the USA,^49^, ^192^ Australia,^23^, ^24^ New Zealand,^26^, ^27^ Japan,^28^, ^193^ and South Korea.^29^ Not all variables were available at all time points for all studies. To be considered, the study had to include at least published data on periodontitis, number of teeth, and edentulism. In addition to dental variables, information on the number of dental visits within the last 12 months, tooth brushing frequency (≥2 times daily), use of interdental cleaning aids, powered toothbrush usage, education, current smoking, and diabetes were extracted from these cohort studies if available. Because information on education, smoking, and diabetes was only partially available in these cohorts, we extracted corresponding data from OECD registries^69^, ^70^ and from the International Diabetes Federation (prevalence of total and unknown diabetes).^73^, ^74^, ^75^ Data on sales volumes of battery and rechargeable power brush handles in Japan, Germany, and the USA has been kindly provided by Procter & Gamble.

All studies reported edentulism, number of teeth, DMF‐T or DMF‐S scores, and the prevalence of CPI scores of 4 or PPD ≥6 mm (severe periodontitis) or CPI scores of 3 + 4 together. Only the two Swedish cohorts reported bone loss. Because the study protocol in the Swedish studies remained the same from wave to wave, we also included these two cohorts. If the mean number of sound teeth was not reported, we calculated it as 28 minus the numbers of missing, filled, and decayed teeth. The prevalence of periodontal health was defined as 100 minus the prevalence of edentulism minus the prevalence of CPI scores 4 (PPD ≥6 mm) and 3 (PPD 4 ‐ 5 mm). When necessary, DMF‐S scores were converted to DMF‐T scores (division by 4.57). If data were not available in tables but in graphs, data were extracted from the graphs.

To provide a comprehensive overview of oral cavity changes, the prevalence of edentulism was included in the DMF‐T scores via inclusion of an additional component: missing teeth in edentates. The idea is as follows: if we assume that in a population of 1000 people, 10% are edentate, they (equals 100 people) account for 2800 missing teeth (assuming that 28 teeth were examined, excluding third molars). To recalculate the DMF‐T the following formula was used: (DMF‐T_dentates_ x N_dentates_ + DMF‐T x N_edentates_)/(N_dentates_ + N_edentates_). For single DMF‐T components (SC; FT and DT), the following formula was applied: SC_dentates_ x N_dentates_/(N_dentates_ + N_edentates_). MT_edentates_ was calculated as 28/(N_dentates_ + N_edentates_). Thus, given a DMF‐T of 18 for dentate subjects with on average 12 filled, 5 missing and 1 decayed teeth, the new DMF‐T components (including the missing teeth component for edentates) would be 10.8 filled teeth, 4.5 missing teeth, and 1.8 decayed teeth in dentate individuals; the 100 edentate individuals would add 2.8 missing teeth for the whole population.

Accordingly, the percentage of edentulous individuals was also considered for the calculation of periodontitis prevalence. In the dental literature information on sound teeth or on periodontal health is rarely reported and even less information is available on DMF‐T scores together with periodontitis and edentulism. This may obscure the effects of population‐wide preventive efforts.

In order to provide a comprehensive picture of oral health, we report periodontal health besides periodontitis to understand, whether the change in periodontitis prevalence resulted in an increase in the number of teeth present. We visualized the trends without accounting for study sizes. Where multiple age stratified data were available (Germany DMS, New Zealand, Spain), data were merged. As the time lags between the repeated waves were quite different, we have converted the changes in the prevalence of edentulism and periodontitis and the number of (sound, filled, decayed) teeth to annual changes. As the recording protocols differed between studies, average numbers of caries‐affected teeth and prevalence of periodontitis cannot be directly compared. All graphs are for descriptive purposes only and are not intended to provide direct comparisons between countries, but they do show trends.

### 8.1 Trends of edentulism, number of teeth, DMF‐T scores and periodontitis in selected countries

Edentulousness can be considered as a robust and reliable proxy for lifetime experience of caries and periodontitis, its sequelae, and dental treatment philosophy. In all cohorts, the prevalence of edentulism has steadily declined, falling from approximately 10–20% to <5% over the respective observation periods (Figure 12A). It is expected that edentulism will almost disappear in more and more countries. The annual decrease in the prevalence of edentulism varied between 0.04 and 0.76%. This trend of decreasing prevalence of edentulism was accompanied by an increase in the number of teeth present in all cohorts (Figure 12B). Regardless of the number of teeth present at first examination, the number of teeth increased almost in parallel by 0.07 to 0.38 teeth/year in most cohorts (Table 5). The highest annual change in the number of teeth present (0.38 teeth/year) was observed in Germany (DMS).

An identical positive trend was observed for the number of sound teeth in all cohorts (Table 5). The annual change in the average number of sound teeth (about 0.2 teeth/year) was independent of the number of sound teeth at the first examination. The trend data for the number of filled teeth showed an inverse time course to that for sound teeth in 6 of the 11 studies. Between 8 and 15 teeth were filled at the first examination and between 3 and 10 teeth at the last examination. The average number of filled teeth decreased (yearly change less than −0.05) in 3 cohorts (New Zealand, England, Sweden Jönköping), remained unchanged (yearly change between −0.05 and 0.05) in 4 cohorts (Australia, Germany, Sweden Dalarna, USA) and increased (yearly change more than 0.05) in 4 cohorts (Spain, Germany SHIP, Japan, South Korea; Figure 13A and Table 5). The average number of sound teeth had increased much more than the average number of filled teeth in most cohorts (Table 5). The change in the average number of decayed teeth showed no clear pattern. Only in six cohorts did the average number of decayed teeth decrease slightly over time, while in the other cohorts, it increased slightly. The annual change in the number of decayed teeth was about 1/10 of the annual change in the number of sound teeth.

In order to understand the temporal interplay between the decreasing prevalence of edentulism and the change in the DMF‐T components, we distinguished whether edentate or dentate individuals contributed to the MT component of the DMF‐T index. Obviously, within populations the MT component of edentulous individuals has decreased and the number of sound teeth has increased over time (Figure 13A, Table 5). Among dentate individuals, the average number of MT or FT decreased in some cohorts (Spain, US), while it remained almost unchanged in other cohorts (Australia, Germany SHIP, Japan, Sweden Dalarna, Sweden Jönköping). These graphs clearly show that the DMFT index only distortedly reflects the DMFT change over time, when the contribution of edentates to the MT component is neglected. In addition, the DMF‐T index does not explicitly focus on sound teeth, so the shift towards a healthier dentition may go unnoticed. A few years ago, Bernabe et al. pointed out that in Western societies the current strategy of dental prophylaxis and treatment is not a causal solution to the caries endemic.^194^ They found that the number of decayed teeth did not decrease and that the number of restorations and/or extractions steadily increased. However, this commentary overlooked the fact that in successive cohorts the annual change in the number of sound teeth exceeded the annual change in the number of carious teeth and that more and more teeth remained obviously sound over time. In parallel, the filling rate declined in many populations, especially in younger populations.^43^ These observations challenge Bernabe's statement that Western dentistry cannot break the caries trend.

The trend for periodontitis is less clear, but it must be noticed that the proportion of edentate individuals significantly decreased while the number of teeth in dentate individuals increased (Figure 13B). So theoretically, more people could be affected by periodontitis, and since these people had more teeth, more teeth were at risk of periodontitis. In contrast to these two trends, which could theoretically lead to a worsening of the periodontal situation, the proportion of healthy or moderately diseased subjects remained unchanged or increased, and the proportion of individuals with severe periodontitis remained more or less the same. It is possible that the decrease in edentulism and the increase in the number of teeth have contributed to the unchanged proportion of people with severe periodontitis. In conclusion, the overall periodontal situation had probably improved only very moderately.

The annual change in the prevalence of periodontally healthy subjects between the first and the last examination was above 1% in four cohorts (New Zealand, Spain, Germany, Sweden Jönköping), whereas in two cohorts there was no notable change (Germany, Sweden Dalarna) and in two cohorts the prevalence of healthy subjects decreased (Australia, Japan) (Table 5, Figure 13B). At the first examination, the proportion of subjects with severe periodontitis predominantly ranged between 5% and 10%. In four cohorts the annual change in the prevalence of severe periodontitis was close to zero, and in no cohort, the prevalence of severe periodontitis decreased by >1% per year. However, changes in the prevalence of edentulism must be considered when interpreting the shift in periodontitis prevalence: in all countries, the prevalence of edentulism decreased and either the prevalence of moderate periodontitis or that of periodontally healthy individuals increased. With the exception of Japan, there was a shift towards more periodontally healthy individuals. Overall, the periodontal situation improved slightly in all populations, although the prevalence of severe periodontitis remained unchanged. These data are not supported by a recent Global Burden of Disease publication, which reported a 23.8% (95% CI: 22.4–24.9) decline in severe periodontitis in high‐income countries over the last 10 to 20 years,^16^ equivalent to a decline of 1 to 2% per year.

### 8.2 Trends in dental determinants in selected countries

We compiled trend data on dental visits within the last 12 months (annual access frequency), tooth brushing frequency (2+ times daily), and interdental cleaning usage for all cohorts (Figure 14). Yearly dental visits were reported by 30 to 90% of study participants and did not change over the past 30 years (Figure 14A). Germans had the highest annual access frequency (90%), while US Americans and South Koreans had the lowest annual access frequency (30 to 40%). The frequency of dental visits seems to depend mainly on whether the annual dental visit is covered by the health insurance or whether it has to be paid out of pocket, at least to a considerable extent. In the other cohorts, 30–60% of participants had seen their dentist within the last 12 months.

In terms of regular tooth brushing (at least twice a day), only one cohort (England) reported no change over time, while all the other cohorts reported a modest or marked increase (Figure 14B). At the last time point, between 65 and 90% of the participants brushed their teeth at least twice a day. Trend data on the use of interdental cleaning devices were available for six cohorts, in three of which (Australia, South Korea, Germany) the proportion of individuals using interdental cleaning aids increased sharply, up to 60% (Figure 14C). Prevalence data on the use of mouth rinses or powered toothbrushes were too sparse to infer a trend. Twice‐daily tooth brushing seems to be firmly entrenched in the minds of the population in all countries, while the use of interdental cleaning aids is increasing strongly. It may be that interdental oral hygiene, whether performed well or poorly, has partly contributed to the improvement of the periodontal situation.

## 9 THE CAUSAL PATHWAY REVISITED

In summary, the proportion of edentulous individuals decreased, and the number of teeth increased in all cohorts. Our key question was whether the change in periodontal status also contributed to the change in the number of teeth, or whether the better tooth retention was mainly due to the change in the caries experience. We assume that the increased number of sound (non‐decayed and non‐filled) teeth mainly contributed to long‐term tooth retention. In addition, we aimed to qualitatively assess the impact of different risk factors, as represented in our causal network, on the increased number of present teeth.

Population‐related measures

- *Education*: The proportion of people with higher education has increased in all cohorts. School and higher education are primarily the responsibility of the state. Higher education can offer people the opportunity to move up in socio‐economic status. Since education is closely related to better oral health knowledge, greater self‐advocacy, better skills and ultimately healthier lifestyle choices, it contributes to improved oral health through multiple channels, both in terms of less caries and periodontitis and more retained teeth.
- *Smoking*: The decline in smoking prevalence is mainly due to government regulation, ranging from taxation to bans and to active encouragement to quit. Smoking is the biggest risk factor for periodontitis, but not for caries.^195^ It is likely that the shift from edentate to dentate individuals has masked the impact of the decline in smoking on periodontitis. The improvement in periodontal health or the shift from severe to moderate periodontitis could be related to the decline in smoking in New Zealand, Spain, Sweden, and the USA.
- *Diabetes*: Diabetes prevalence increased in all cohorts. Although the proportion of people with undiagnosed diabetes decreased slightly, diabetics with poor metabolic control drive up periodontitis prevalence. The increase in diabetes prevalence may have had a significant impact on the unchanged prevalence of severe periodontitis.
- *Dental insurance system*: The dental insurance system is likely to have a significant impact on the actions of the dentist. We do not currently know whether one insurance system works better than another in terms of periodontal treatment and retention of periodontally diseased teeth.
- *Oral care industry*: Fluoridated toothpaste was introduced by the industry 30–40 years ago and has a market penetration of 80%–90% in high‐income countries. In addition to public health measures such as salt or drinking water fluoridation, the widespread use of toothpaste has led to a decline in caries experience. In addition, regular tooth brushing is associated with a greater reduction in caries lesions, which is thought to be due to the oral bathing of teeth with fluorides rather than the removal of plaque.^196^ It is likely that the population‐wide introduction of fluoridated toothpaste and its twice‐daily use favored the continued increase in sound teeth and the decrease in filled teeth and had the greatest impact on dental health. In summary, the higher number of retained teeth was associated with the continued use of fluoridated toothpastes.
- *Interdental cleaning aids and powered toothbrushes*: Up to 60% of people use interdental cleaning aids and up to 50% use powered toothbrushes. Whether the increased use of these oral hygiene aids is due to individual patient instruction in the dental practice or to advertising by the oral care industry cannot be assessed. In summary, better tooth retention is probably also related to greater use of interdental cleaning aids and powered toothbrushes.
- *Industry advertisement*: We do not know how much the industry spends on advertising and whether the budget has increased in recent years. All we know is that the budget for oral care products in the US and Germany has increased by about 15% in the last 6–7 years. In summary, we do is not enough information available to estimate the impact of industrial advertising.

Individual dental interventions

- *Periodontal care*: As no reduction in severe periodontitis was observed in most cohorts, it can only be assumed that periodontal interventions probably did not contribute very much to the higher tooth retention rate. The published, albeit sparse, literature suggests that prophylactic measures did not contribute to increased tooth retention. There is also limited information on the shift in the extraction paradigm.^171^ In summary, the impact of periodontal treatment on tooth retention in the general population is likely to be small.
- *Oral hygiene instructions*: The use of powered toothbrushes and interdental cleaning aids is beneficial for tooth retention. The extent to which individual oral hygiene instruction, in addition to industry advertising, has contributed to the increase in use is unknown.^6^ Most of the improvement is likely to be due to industry advertising, but the interaction between industry advertising and individual actions cannot be broken down from this data.

Contrary to the statement, “that in high‐income countries, the current treatment‐dominated and technology‐focused system of oral health care is trapped in an interventionist cycle failing to tackle the underlying causes of diseases and not meeting the needs of large proportions of the population”,^5^ we found an improvement in overall oral health in terms of caries burden, while periodontal conditions did not change or changed only slightly.

## CONFLICT OF INTEREST STATEMENT

All authors declare that they have no conflicts of interest in connection with this article.

## Data Availability

Generally, data sharing is not applicable to this article as no new data were created or analyzed in this study. Regarding data provided by Procter & Gamble on volume of sold items of powered toothbrushes and of mouth wash, author elects to not share data. Data from the National Health and Nutrition Examination Survey are openly available in a public repository that does not issue DOIs (https://wwwn.cdc.gov/nchs/nhanes/Default.aspx).
